# Targeting EDEM protects against ER stress and improves development and survival in *C*. *elegans*

**DOI:** 10.1371/journal.pgen.1010069

**Published:** 2022-02-22

**Authors:** Simona Ghenea, Marioara Chiritoiu, Robi Tacutu, Antonio Miranda-Vizuete, Stefana Maria Petrescu

**Affiliations:** 1 Department of Molecular Cell Biology, Institute of Biochemistry of the Romanian Academy, Bucharest, Romania; 2 Department of Bioinformatics and Structural Biochemistry, Institute of Biochemistry, Romanian Academy, Bucharest, Romania; 3 Redox Homeostasis Group, Instituto de Biomedicina de Sevilla (IBIS), Hospital Universitario Virgen del Rocio/CSIC/Universidad de Sevilla, Sevilla, Spain; The University of Texas Health Science Center at Houston, UNITED STATES

## Abstract

EDEM-1, EDEM-2 and EDEM-3 are key players for the quality control of newly synthesized proteins in the endoplasmic reticulum (ER) by accelerating disposal and degradation of misfolded proteins through ER Associated Degradation (ERAD). Although many previous studies reported the role of individual ERAD components especially in cell-based systems, still little is known about the consequences of ERAD dysfunction under physiological and ER stress conditions in the context of a multicellular organism. Here we report the first individual and combined characterization and functional interplay of EDEM proteins in *Caenorhabditis elegans* using single, double, and triple mutant combinations. We found that EDEM-2 has a major role in the clearance of misfolded proteins from ER under physiological conditions, whereas EDEM-1 and EDEM-3 roles become prominent under acute ER stress. In contrast to SEL-1 loss, the loss of EDEMs in an intact organism induces only a modest ER stress under physiological conditions. In addition, chronic impairment of EDEM functioning attenuated both XBP-1 activation and up-regulation of the stress chaperone GRP78/BiP, in response to acute ER stress. We also show that pre-conditioning to EDEM loss in acute ER stress restores ER homeostasis and promotes survival by activating ER hormesis. We propose a novel role for EDEM in fine-tuning the ER stress responsiveness that affects ER homeostasis and survival.

## Author summary

ER stress and UPR^ER^ malfunctions have been implicated in the pathogenesis of neurodegeneration, metabolic and inflammatory diseases as well as tumor progression and diabetes, whereby disturbed ER homeostasis negatively influences the pathology of the disease. Under ER stress conditions, the cells either activate UPR^ER^-dependent cytoprotective mechanisms when ER stress is at subtoxic levels or, in case of an excessive ER stress, the cytotoxic response stimulates cell death. Here, we used *Caenorhabditis elegans* to study the cellular responses to ER stress at organismal level. We show that EDEMs respond differently to ER stress stimuli, and moreover, EDEMs deficiencies activate an XBP-1 independent adaptive program to promote organism survival under acute ER stress. Corroborated with the fact that loss of EDEM-2 and EDEM-3 induces resistance to acute ER stress in an intact organism, our data implicate EDEM proteins in a broader response to ER stress than previously established, which opens a new avenue for understanding the regulation of ER stress with implications for clinical and therapeutic investigations.

## Introduction

Cells respond to stress stimuli by modulating a wide range of cellular processes to promote survival of the organism. Failure to activate an efficient response to stress disturbs organelle homeostasis. Impairment of organelle homeostasis triggers activation of compensatory stress responses such as ER unfolded protein response (UPR^ER^), mitochondrial unfolded protein response (UPR^mt^), peroxisomal quality control system and autophagy aiming to restore their normal function [1,2]. Maintaining ER homeostasis is critical for cell survival because ER is the place of protein and lipid synthesis and plays a major role in the regulation of Ca signalling. Misfolded or incompletely folded proteins are recognized by the ER quality control surveillance system and removed from ER to be degraded by the proteasome in a process called ER-associated degradation (ERAD) [3–5]. Dysfunctions in ERAD lead to ER stress by overload with defective proteins, which further disrupts ER homeostasis. In humans, ER stress has been associated with a wide range of pathologies such as age-related maladies, cancer, metabolic diseases and inflammation [6–9].

ERAD is a multi-step process, relying on the function of protein complexes with role in recognition, retrotranslocation and ubiquitination of misfolded proteins for subsequent proteasomal degradation [10]. The best understood is the ERAD of terminally misfolded glycoproteins (gpERAD) in which the substrate recognition is based on extensive trimming of mannose residues from the N-glycan attached to the polypeptide chain. Both in yeasts and mammals, the presence of a glycan with less than 9 mannose residues indicates that the protein is misfolded and should be degraded [11,12]. In yeasts, the ER mannosidase MnsI has a central role in the initiation of mannose trimming as it catalyses the removal of the first mannose residue, whereas the α1,2-mannosidase, Htm1, performs the subsequent trimming to generate the degradation signal. Higher eukaryotes possess three orthologues for Htm1, the ER degradation-enhancing α-mannosidase 1, 2 and 3 (EDEM1, EDEM2 and EDEM3) proteins, which have been implicated in the degradation of both soluble and transmembrane substrates of gpERAD [13–16]. All three proteins participate in mannose trimming of gpERAD substrates, exhibiting mannosidase activity [16–19]. In mammals, both ERmanI (the mammalian orthologue of MnsI) and EDEM2 can initiate mannose trimming [18,20]. However, a prominent role of ERmanI in mannose trimming is under debate since it has been suggested that, in contrast to yeast, the ERmanI has only a minor role in mammalian gpERAD. Instead, mammalian EDEM2 has been shown to play the central role in the initiation of mannose trimming, whereas EDEM3 and EDEM1 perform the subsequent trimmings [18]. Recent studies indicate that EDEM1 and EDEM2 are also able to mediate degradation of ERAD substrates in a glycan-independent manner [21–23]. An interplay between gpERAD and non-gpERAD during clearance of misfolded glycoproteins is supported by the observation that although degradation of severely misfolded glycoproteins is impaired at early chase periods, they are ultimately cleared from ER by an unknown process that restores the ER homeostasis [24].

How misfolded proteins from ER are processed and the impact of ERAD disruption in the context of a whole organism is still elusive. In this study we used genetic analysis to investigate the mechanism that links ERAD defects to cellular responses. We specifically investigated the role of EDEM-1, EDEM-2, and EDEM-3 in *C*. *elegans* development and aging. We found that loss of EDEM-2 causes pleiotropic defects that are not further aggravated by EDEM-1 and/or EDEM-3 loss under physiological conditions, indicating a major role for EDEM-2 in targeting misfolded proteins for proteasomal degradation under physiological conditions. Moreover, EDEMs respond differently to ER stress stimuli and both EDEM-1 and EDEM-3 functions become evident under acute ER stress. In addition, our data provide the first evidence that preconditioning to *edem* loss activates a hormetic XBP-1 independent adaptive program that enhances ER stress proteotoxic responses to promote organism survival under acute ER stress.

## Results

### *edem-1*, *edem-2*, and *edem-3* expression patterns in *C*. *elegans*

**Fig 1:**
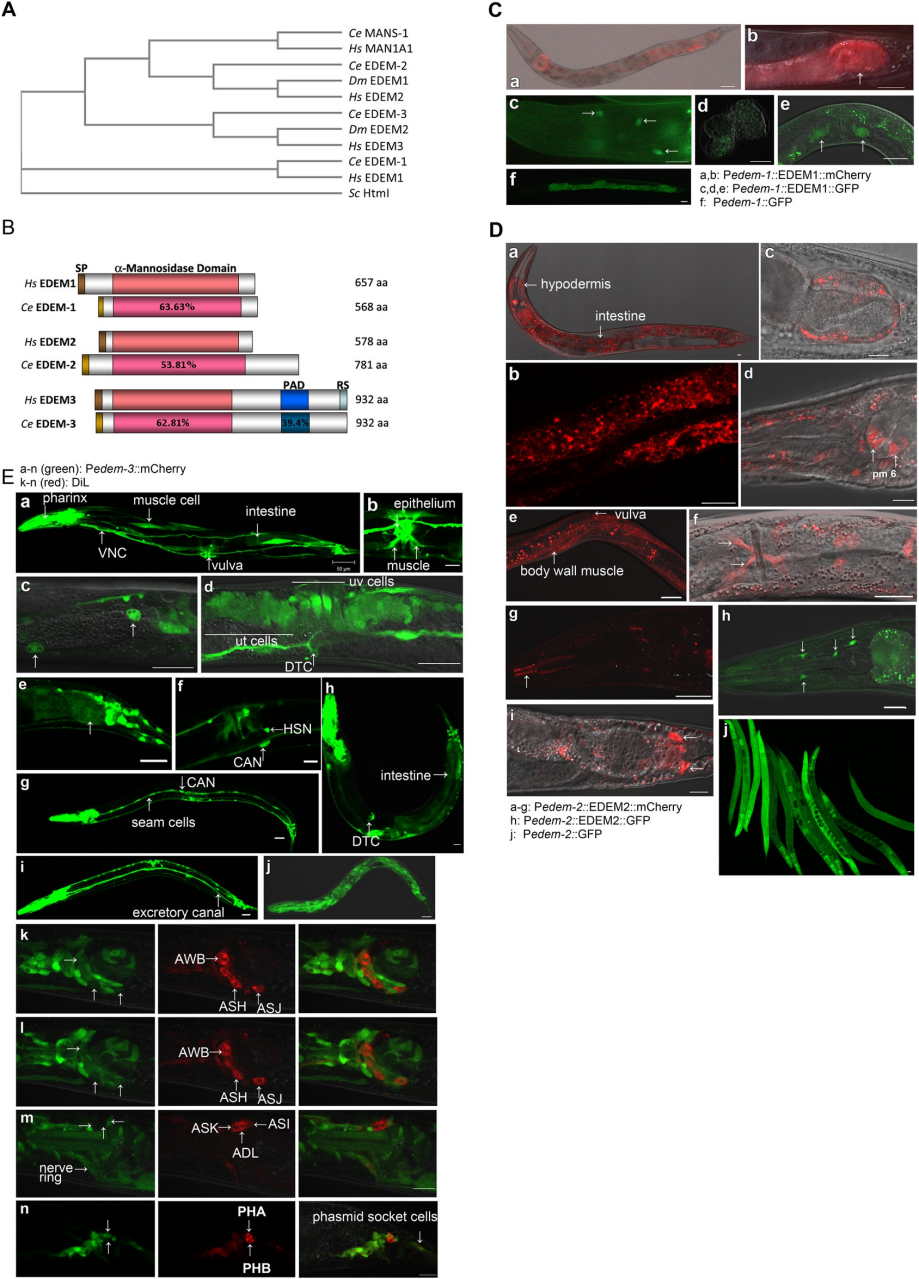
Expression pattern of *edem-1*, *edem-2*, and *edem-3* genes. **(A)** Phylogenetic analysis of the human (Hs—*Homo sapiens*), fly (Dm—*Drosophila melanogaster*), worm (Ce—*Caenorhabditis elegans*) and yeast (Sc—Saccharomyces cerevisiae) orthologues of the ER α-mannosidase proteins. The alignment was made with Clustal Omega software [71]. **(B)** Schematic organization and pairwise sequence similarity (% identity) between conserved regions of *C*. *elegans* (Ce) and human (Hs) EDEM proteins. Sequence identity between domains is shown based on Clustal W alignment [72]. SP denotes signal peptide, PAD- protease associated domain, RS- ER retention signal. **(C)** Constitutive expression of *edem-1* reporter was detected in intestine (a), hindgut (arrow in b) (visualized with P*edem-1*::EDEM-1::mCherry) and head neurons (visualized with P*edem-1*::EDEM-1::GFP) (arrows in c). Upon the ER stress, P*edem-1*::GFP was detected in embryos (d), uterus (e), and upregulated in intestine (f). **(D)** Constitutive expression of P*edem-2*::EDEM-2::mCherry was detected in hypodermis (a), intestine (a, and magnification in b), hindgut (c), pm6 cells of pharynx (d) body wall muscle cells (e), vulva muscle (f), pharyngeal epithelial cells (g), neurons in the head (h, visualized with P*edem-2*::EDEM-2::GFP), and tail neurons (i). P*edem-2*::GFP was almost ubiquitously expressed upon induction of ER stress by heat shock (j). **(E)** P*edem-3*::GFP was constitutively expressed in pharynx, nervous system, body wall muscle cells (a), vulval muscle and epithelium (b), coelomocytes (c) uterus (ut and uv cells) and DTC (distal tip cells) (d), hindgut (arrow) and tail structures (e), HSN neurons (f) and intestine of L1-L2 larvae (j). Upon induction of ER stress, P*edem-3*::GFP was detected in seam cells (g), intestine (h), and excretory canals (i). In (k-m) are shown three focal planes of a P*edem-3*::GFP young adult stained with DiI. The dye stained AWB, ASH, ASJ, ASK, ADL and ASI neurons, and the P*edem-3*::GFP expression is seen in AWB, ASK (weak expression), ASJ and ASI but not in ASK and ADL neurons. The P*edem-3*::GFP is expressed in the PHA but not in PHB phasmid neurons filled with DiI (n). The micrographs were obtained by confocal optics. Scale bars: 10 μm.

The *C*. *elegans* genome encodes all three orthologues of the ER α-mannosidase-like subgroup of glycosyl hydrolase family 47, which we hereafter referred to as *C*. *elegans edem-1* (for C47E12.3), *edem-2* (for F10C2.5) and *edem-3* (for ZC506.1) (Fig 1A). The putative *C*. *elegans* EDEM-1, EDEM-2 and EDEM-3 proteins share 46, 39 and 41% amino acid identity and present similar structural organization as their human orthologues, with a notable difference, EDEM-3 does not have the conserved sequence for ER-retention signal (RS) (Fig 1B). Outside of the α-mannosidase domain there is little amino acid identity between the *C*. *elegans* EDEM sequences and human counterparts. To elucidate their role in a multicellular organism we first investigated the expression pattern of *edem-1*, *edem-2* and *edem-3* in *C*. *elegans* wild type (WT) animals, using transcriptional and translational fluorescent reporters. We found that both *edem-1* and *edem-2* are constitutively expressed in the gut, with stronger expression in anterior and posterior gut cells (Fig 1Ca and 1Da-b). EDEM-1 was also detected in hindgut (Fig 1Cb) and a few neurons in the head (Fig 1Cc), whereas EDEM-2 was detected in hypodermis (Fig 1Da), hindgut (Fig 1Dc), pm6 muscle cells of the pharynx (Fig 1Dd), body wall muscle (Fig 1De), vulval muscle (Fig 1Df), pharyngeal epithelial cells (Fig 1Dg), and in a few neurons in the head (Fig 1Dh) and tail (Fig 1Di). In addition, under ER stress conditions, P*edem-1*::GFP was detected in the embryos (Fig 1Cd) and uterus (Fig 1Ce), whereas P*edem-2*::GFP was ubiquitously detected (Fig 1Dj). Microinjection of the P*edem-3*::EDEM-3::GFP construct in WT animals produced uncoordinated progeny that failed to generate stably transmitting transgenic line, suggesting a possible toxic effect caused by overexpression, misfolding or subcellular mislocalization of the EDEM-3::GFP fusion protein. Therefore *edem-3* expression was established using the transcriptional GFP reporter (Fig 1E). P*edem-3*::GFP expression was detected in the pharynx, nervous system, body wall muscle (mosaic expression) (Fig 1Ea), coelomocytes (Fig 1Ec), hindgut and tail structures (Fig 1Ee), sensory neurons (Fig 1Ek-n), and CAN neurons (visible in Fig 1Ef and 1Eg). In young adults the expression became apparent in vulva muscle and vulval epithelium (Fig 1Eb), uterus (Fig 1Ed), distal tip cells (Fig 1Ed), and hermaphrodite specific neuron (HSN) (Fig 1Ef). P*edem-3*::GFP expression in the gut was transiently activated in L1 and L2 larvae (Fig 1Ej), then faded and reappeared in older animals (mosaic expression) (Fig 1Ea). In addition, P*edem-3*::GFP became visible in seam cells (Fig 1Eg), intestine (Fig 1Eh), and excretory canals (Fig 1Ei) upon ER stress. We conclude that, although the expression of each *edem* (*edem-1*, *edem-2* and *edem-3*) is constitutively detected in specific cells and tissues under physiological conditions, their expression can be upregulated in additional cells and tissues upon ER stress.

### *C*. *elegans* EDEMs share functional similarities with mammalian orthologues

**Fig 2:**
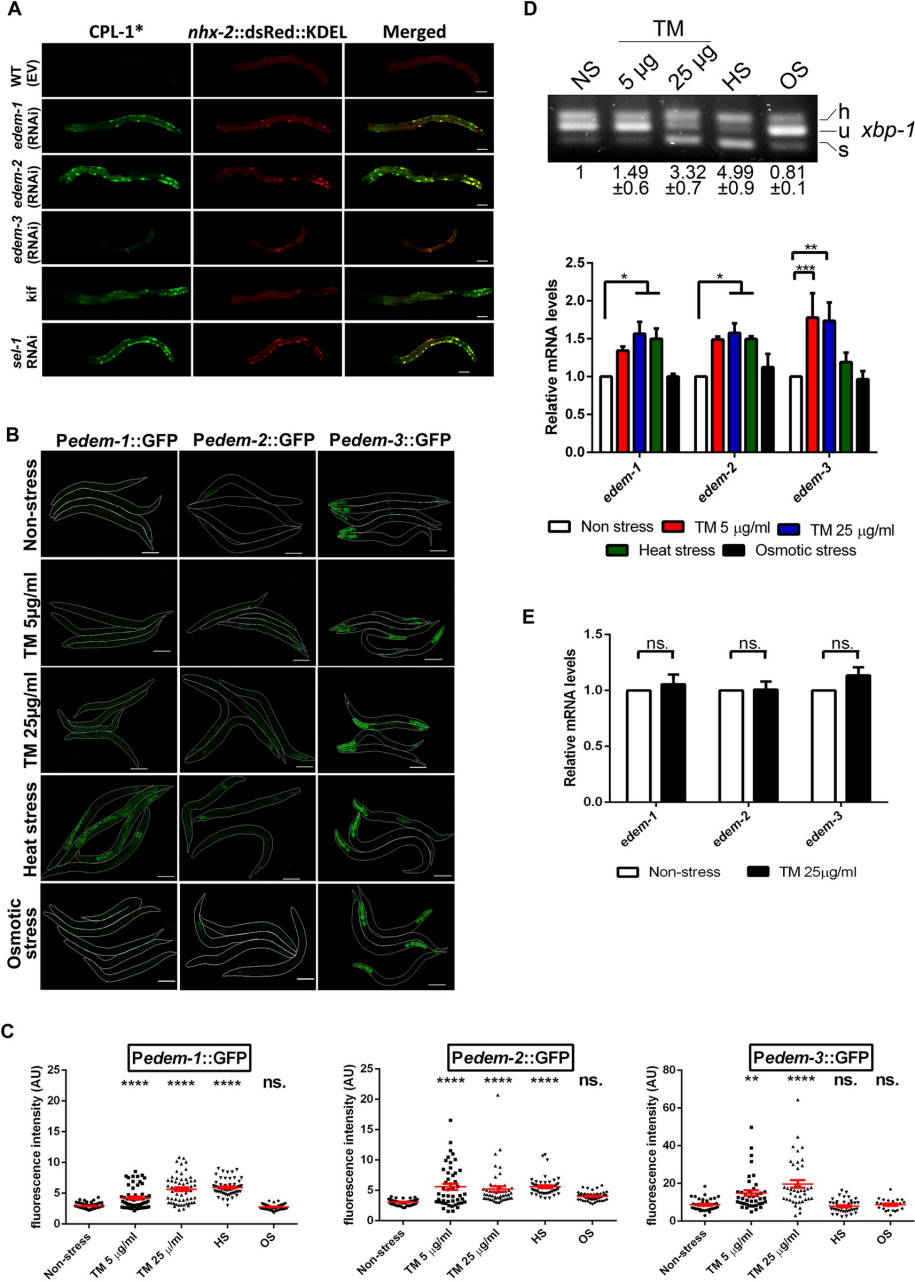
*C*. *elegans edem* genes have conserved functions in the clearance of misfolded proteins from ER. **(A)** RNAi downregulation of *edem* triggered accumulation of CPL-1* in intestinal cells. CPL-1* localized to ER, visualized with the ER marker *nhx-2*::dsRed::KDEL (*nhx-2*—Na/H eXchanger—is expressed in the apical membranes of the intestinal cells), in *edem* and *sel-1* silenced animals. For kifunensine treatment (Kif) animals were kept on 30mM kifunensine for 24h before imaging. Scale bar: 20 μm. **(B-C)** P*edem*::GFP transgenes responded to the ER stress treatments: 5 μg/ml and 25 μg/ml tunicamycin for 6 h, heat stress (35°C for 4.5 h) but not to osmotic stress (200 mM NaCl). Animals were imaged in **(B)** and the fluorescence was quantified in **(C)**. The worms are outlined for better visualization. Images were acquired with identical confocal settings for each reporter. Scale bar: 20 μm. Dots in scatter grams represent mean fluorescence/μm^2^ x 1000 in arbitrary units (AU). The red bars indicate the average ±SEM. **(D)** Young adult WT animals non-treated (NS) or subjected to the treatments presented in (B) were collected for RNA isolation and reverse transcription. The resulting cDNAs were used to assess both *xbp-1* mRNA splicing ratio by PCR analysis and *edem* relative mRNA levels by qPCR. The upper panel shows the forms of *xbp-1* mRNA detected in the agarose gel (h-hybrid, u-unspliced and s-spliced). Band intensity of samples was measured and the mean values ±SD of the ratio spliced/unspliced *xbp-1* normalized to Non-stress sample are shown beneath each band. The lower panel shows the qPCR measurements of *edem* relative mRNA levels. The mRNA level under Non-stress conditions was set to value = 1 and the treatment values indicate the fold change ± SEM compared to Non-stress value. (n = 4, two way ANOVA with Dunnett’s multiple comparisons test). **(E)** Upregulation of *edem* expression by tunicamycin is dependant on IRE-1. Quantitative RT-PCR measurements of *edem* mRNA levels in *ire-1(v33)* mutants without or with tunicamycin treatment (25μg/ml tunicamycin for 6h). (n = 3 independent experiments, two way ANOVA with Bonferroni’s post test). *P<0.05; **P<0.01; ***P<0.001; ****P<0.0001; ns, not significant.

Overexpression of any EDEM protein in mammalian cell culture was shown to accelerate translocation from the ER and proteasomal degradation of the terminally misfolded glycoproteins [13–16,25]. To test whether EDEM proteins have conserved functions in *C*. *elegans* we investigated the impact of EDEM downregulation by RNAi on the degradation of the glycoprotein pro-cathepsin L (CPL-1), a well-established ERAD substrate, in intestinal cells [26]. The misfolded CPL-1(W32A Y35A)::YFP (CPL-1*) protein is degraded by the proteasome in WT control animals and is retained in ER when ERAD is impaired [26]. CPL-1* was not detected in control animals fed with RNAi empty vector, WT (EV), whereas it accumulated in abnormal enlargements of ER in animals with downregulated *edem-1*, *edem-2* as well as *sel-1*, which we used as positive control for dysfunctional ERAD (Fig 2A). CPL-1* also accumulated in the intestine of WT animals treated with kifunensine, a chemical that inhibits all ER mannosidases, confirming the capacity of kifunensine to inhibit ERAD in *C*. *elegans* as in mammals (Fig 2A). In contrast, degradation of CPL-1* occurred in animals fed with *edem-3* RNAi (Fig 2A), which might suggest that either EDEM-3 does not have an essential function in the degradation of misfolded CPL-1* or EDEM-3 has a redundant role with EDEM-1 and/or EDEM-2 in CPL-1* degradation. qPCR analysis showed only a partial inactivation of *edem-3* (S1A Fig), probably due to the major expression of *edem-3* in the nervous system, which is resistant to RNAi treatment. Note that the fluorescence of stress granules, which is known to accumulate in the intestine during cellular stress, is negligible (S1C Fig), and it does not significantly increase the CPL-1* fluorescence.

The canonical UPR^ER^ is conserved in *C*. *elegans* and the ER stress activates the UPR^ER^ in a similar way as it occurs in mammals [27–29]. We found that the ER stressors tunicamycin (inhibitor of N-linked glycosylation) and heat shock (inducer of protein misfolding by disrupting the Van der Waals forces) significantly increased the expression of P*edem-1*::GFP and P*edem-2*::GFP reporters as shown by both fluorescence microscopy and Western blotting (Figs 2B, 2C and S1B). The P*edem-3*::GFP expression was only increased by tunicamycin treatments. In contrast, there was no increase in P*edem*::GFP fluorescence upon osmotic stress treatments which induce cytosolic protein aggregation (Figs 2B, 2C and S1B).

The activation of *edem* genes by the ER stressors was confirmed by quantifying the endogenous *edem* mRNA levels through qRT-PCR. In these experiments, the efficacy of the applied stressors to induce ER stress and activate the IRE-1 pathway was evaluated by monitoring *xbp-1* mRNA splicing (Fig 2D upper panel). An active form of the transcription factor XBP-1 (XBP-1s), which induces expression of many downstream ERAD genes, is generated by IRE-1-mediated unconventional splicing of *xbp-1* mRNA [30]. All three *edem* genes responded to tunicamycin treatment by increasing the mRNA level, but only the mRNA level of *edem-1* and *edem-2* significantly increased in response to heat stress (Fig 2D lower panel). No difference in mRNA levels was seen upon osmotic stress treatments suggesting that *edem* genes are specifically activated by ER stress. Next, to confirm that in *C*. *elegans* activation of *edem* genes by ER stress is IRE-1-dependent, as in mammals, we measured *edem* mRNA levels in IRE-1-deficient worms exposed to a high dose of tunicamycin. As expected, the upregulation of *edem* mRNAs by tunicamycin was abolished in *ire-1* mutants (Fig 2E). Taken together, sequence analysis, ER accumulation of the misfolded CPL-1* upon EDEM depletion, induction of gene expression by ER stressors and control of expression by the IRE-1/XBP-1 pathway indicate that the *C*. *elegans* EDEMs are required for the quality control in the ER and have an evolutionarily conserved role in disposal of misfolded proteins.

### *edem-2* mutants exhibit pleiotropic defects under physiological and mild ER stress conditions

**Fig 3:**
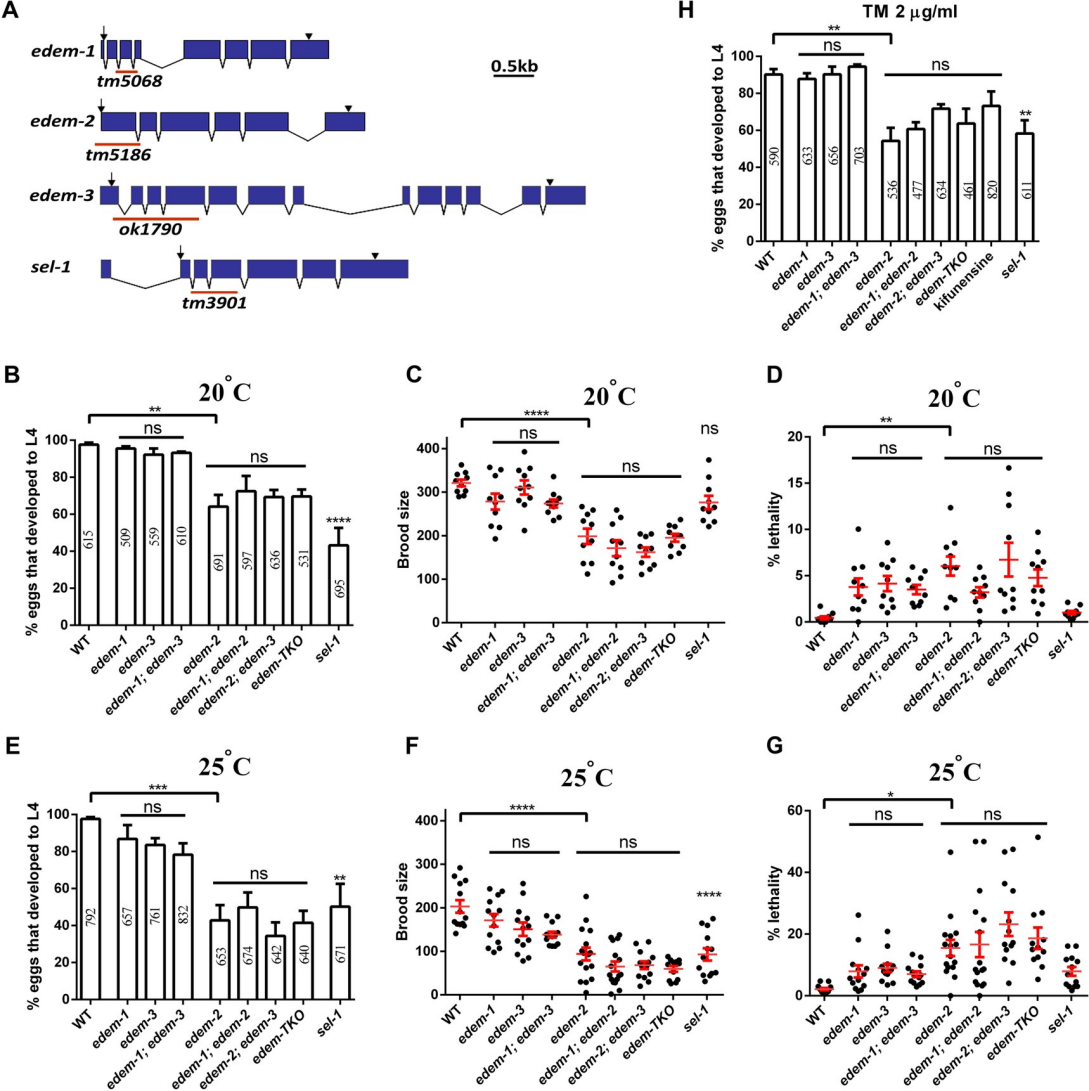
Phenotypes of *edem* mutants under physiological conditions. **(A)** Genomic organization of *edem-1*, *edem-2* and *edem-3* genes. Exons are indicated by blue boxes and introns by lines; the ATG initiation (arrow) and termination codons (arrowheads) are indicated. The lines underneath indicate the position of deletion in the corresponding allele. **(B)** Growth rate of the indicated strains at 20°C. Eggs were laid on the plates and the percentage of eggs that reached L4 stage 52h later was scored. Each strain was scored on three replicate plates in four independent experiments. **(C)** Brood size of the indicated strains at 20°C. Each point represents the total brood of one hermaphrodite. **(D)** Percentage lethality of the indicated strains grown at 20°C. Each point represents % lethality of progenies derived from one hermaphrodite. Lethality encompasses both embryonic and larval arrest. **(E)** Growth rate of the indicated strains at 25°C. Eggs were laid on the plates and the percentage of eggs that reached L4 stage 48h later was scored. Each strain was scored on three replicate plates in five independent experiments. **(F)** Brood size of the indicated strains at 25°C. Each point represents the total brood of one hermaphrodite. **(G)** Percentage lethality of the indicated strains grown at 25°C. Each point represents % lethality of progenies derived from one hermaphrodite. Lethality encompasses both embryonic and larval arrest. **(H)** Growth rate under mild tunicamycin stress. Eggs were laid on the plates with 2 μg/ml tunicamycin and the percentage of eggs that developed into L4 larvae after 3 days was scored. Each strain was scored on three replicate plates in four independent experiments. The red bars in C-D and F-G indicate the mean ±SEM. The values inside the columns in B, E and H represent the total number of eggs analyzed. *P<0.05; **P<0.01; ***P<0.001; ****P<0.0001; ns, not significant.

Next, we analyzed various phenotypes, including morphological defects, growth rate, fertility and lethality of the following *edem* deletion alleles: *edem-1(tm5068)*, *edem-2(tm5186)* and *edem-3(ok1790)*, as well as *sel-1(tm3901)* (Fig 3A). The *edem-1(tm5068)* is likely a null allele since the deletion causes a frameshift from nucleotide 91 that removes almost the entire amino acid sequence. The deletion in *edem-2(tm5186)* removes the TATA box and the nucleotides encoding the first 148 amino acids including the ER targeting signal and therefore is likely a null allele. The deletion in *edem-3(ok1748)* causes an early frameshift in the reading frame, the gene encoding only for an incomplete ER targeting signal and hence, *edem-3(ok1790)* is also likely a null allele. The *sel-1(tm3901)* is a deletion allele that removes the sequence encoding for the first three SEL-1-like repeats.

**Fig 4:**
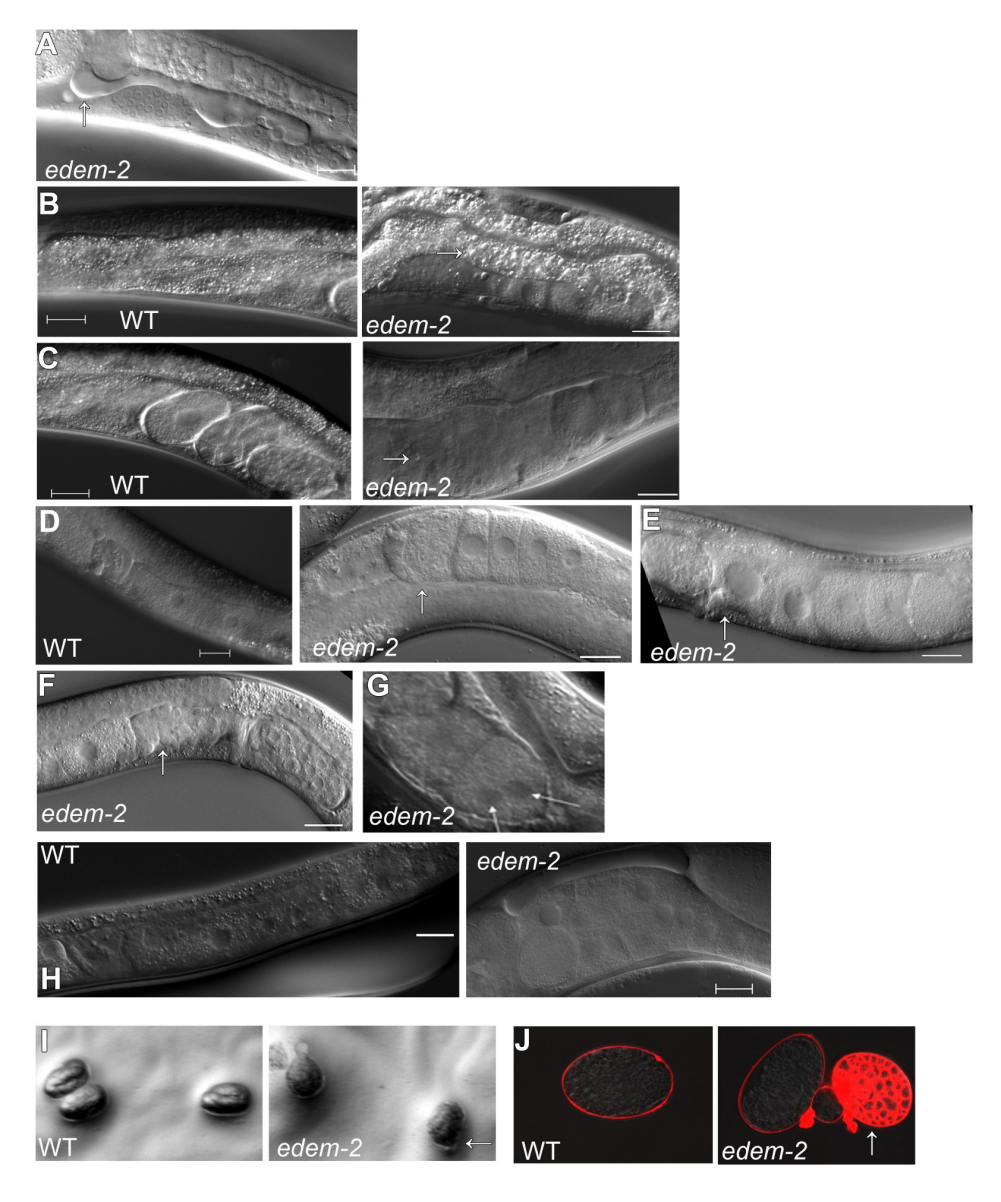
Morphological defects of *edem-2* mutants. Representative Nomarski images of *edem-2* mutant animals showing: **(A)** phase liquid accumulation into the body cavity, **(B)** large vesicles in intestine, **(C)** torn embryos in the uterus**, (D)** torn oocytes in the proximal gonad**, (E)** unfertilized oocytes with a large nucleus in the uterus, **(F)** undeveloped uterus, **(G)** cytokinesis defects (2 cells embryo with a binucleated cell), and **(H)** oocytes accumulation in proximal gonad reflecting defective ovulation. **(I)** Nomarski images of WT embryos and *edem-2* herniated embryos. Arrows indicate the position where chitin is broken, and the cytoplasm of the embryo is extruded. Images were acquired with Zeiss stereo microscope Discovery V20. **(J)** WT and *edem-2* embryos stained with FM4-64 dye. While in WT embryo the chitin formed an effective barrier that blocked dye entrance, the dye penetrated the chitin of *edem-2* embryos. Arrow points to an embryo in which the dye penetrated and stained the cell membranes. Scale bar: 20 μm.

We found that under physiological conditions (20° C) each EDEM has a particular role for ER homeostasis and implicitly for animal development and survival. Homozygous *edem-1* and *edem-3* mutants did not show visible morphological defects, had a growth rate and fertility similar to WT animals (Fig 3B and 3C) and presented low lethality (Fig 3D). The lethality was due exclusively to embryonic lethality in the case of *edem-1* mutants and to both embryonic and L2 larval arrest (which eventually dies) in the case of *edem-3* mutants. By contrast, homozygous *edem-2* mutants exhibited more obvious defects that were temperature and age variable. *edem-2* mutants developed slower than WT animals (Fig 3B) and became slow moving and slightly uncoordinated as they aged. Moreover, *edem-2* mutants had lower broods than WT animals (Fig 3C) and showed a slightly but significantly increased lethality, (Fig 3D). We also found that 12.5% of *edem-2* hermaphrodites (4 out of 32) were completely sterile. An increase in temperature (25° C) slightly amplified the basal defects of growth, lethality and fertility for all *edem* mutants indicating that their loss is making the worms more sensitive to mild ER stress (Fig 3E–3G). Even under these conditions the role of EDEM-2 on growth, lethality and fertility was more prominent than EDEM-1 and EDEM-3 roles. The most obvious defects of *edem-2* mutants were phase liquid accumulation into the body cavity (Fig 4A), presence of large vesicles in the intestine (Fig 4B), torn embryos in the uterus (Fig 4C) and gonad and uterus defects (Fig 4D–4H). These defects had a high penetrance at 25° C but they were also obvious at 20° C. Moreover, *edem-2* hermaphrodites laid a slightly increased number of dead embryos during the second half of the fertility period, which were often smaller than WT embryos (indicative of dysfunctional ovulation) or did not maintain the oval shape. Some of the embryos were herniated (Fig 4I) and permeable to the lipophilic dye FM4-64 (Fig 4J), consistent with the possibility of a weak eggshell caused by defects in either synthesis or secretion of eggshell components by the hypodermal cells. Secretion and cytokinesis defects were also reported for inactivation of OST complex components [31]. Therefore, *edem-2* mutants have a high degree of pleiotropy, suggesting that EDEM-2 plays a more prominent role in maintaining proteostasis, whereas EDEM-1 and EDEM-3 have only minor roles under physiological and mild ER stress conditions.

### The defects of *edem-2* mutants are not enhanced by the loss of EDEM-1 or EDEM-3 proteins under physiological or mild ER stress conditions

To test whether EDEM proteins have overlapping functions *in vivo* we generated double and triple *edem* mutant strains. We found that addition of *edem-1* and/or *edem-3* mutations did not obviously alter the morphological defects of *edem-2* mutants either at 20° C or at 25° C. Consistent with these observations, *edem-1; edem-2* and *edem-2; edem-3* double mutant animals as well as *edem-1; edem-2; edem-3* triple mutants (*edem-TKO*, for short) had similar growth rate, brood size and lethality as *edem-2* single mutants under both physiological (Fig 3B–3D) and mild ER stress (Fig 3E–3G) conditions. *edem-1; edem-3* double mutants did not show synergistic or additive defects on the phenotypes of the respective single mutants under physiological (Fig 3B–3D) or mild ER stress (Fig 3E–3G) conditions, neither phenocopied *edem-2* morphological defects. Similar effects were observed upon ER stress induced by low concentration of tunicamycin (2 μg/ml). Tunicamycin had no visible effect on *edem-1* and *edem-3* single mutants, but it decreased the growth rate of *edem-2* and *sel-1* mutants, (Fig 3H). Addition of *edem-1* and/or *edem-3* mutations only slightly altered the developmental delay of *edem-2* mutants induced by tunicamycin, although not statistically significant (Fig 3H). Collectively, these results suggest that under physiological conditions or mild ER stress, all three *edem* genes might be involved in common genetic pathways that affect growth, lethality and fertility. Our findings in an animal model are in accordance with results from cell culture where EDEM2 was shown to be the main mannosidase that initiates mannose trimming from denatured glycoprotein, whereas EDEM3 and EDEM1 complete mannose trimming, a process that generates degradation signal for misfolded proteins[18].

### EDEM-1 and EDEM-3 are required for ER homeostasis under acute ER stress

**Fig 5:**
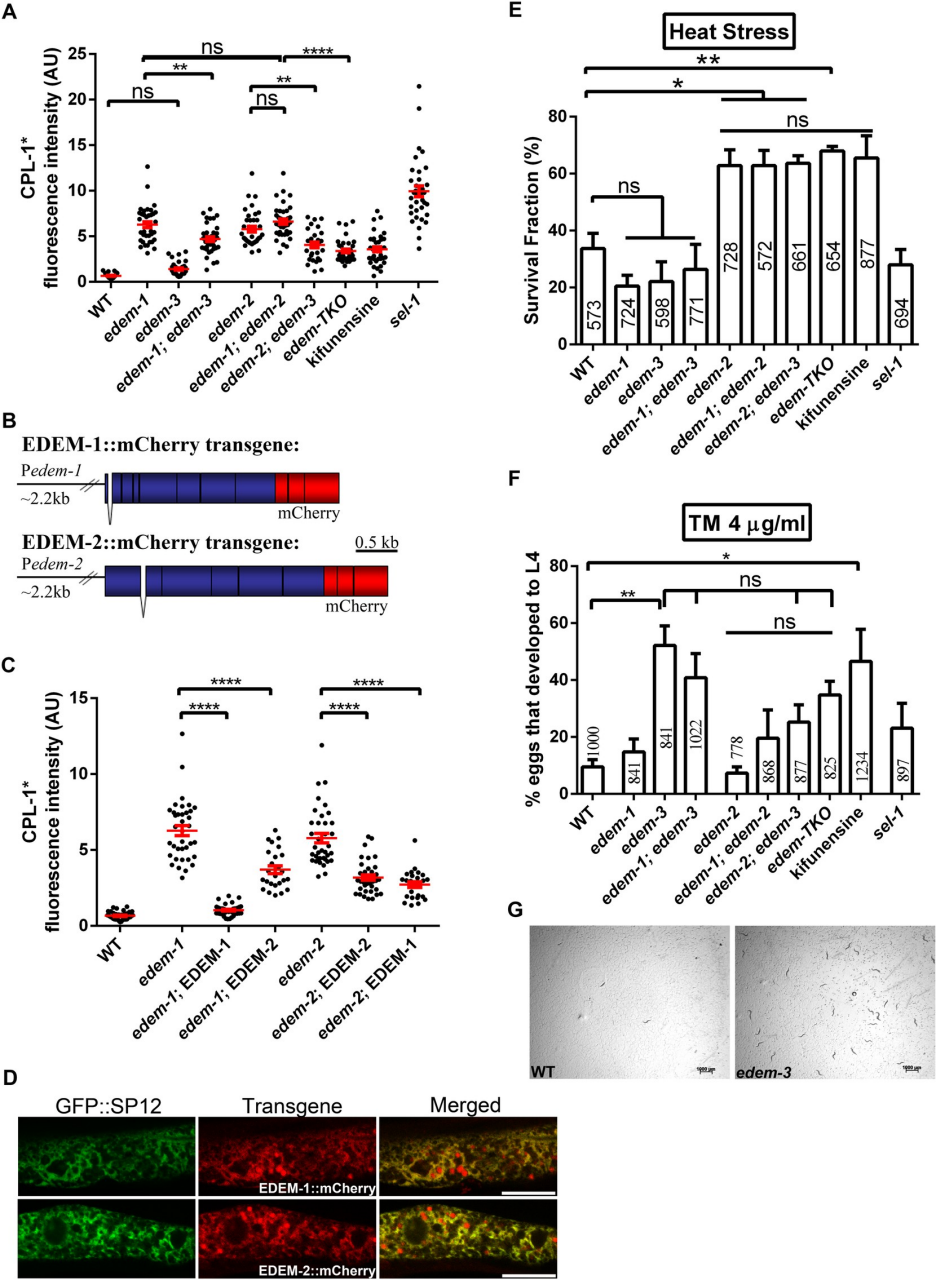
*edem* mutants respond differently to ER stress. **(A)** Fluorescence intensities of indicated strains carrying the CPL-1* transgene. Dots in scatter gram represent mean fluorescence/μm^2^ x 1000 in arbitrary units (AU). **(B)** Schematic structure of P*edem-1*::EDEM-1::mCherry and P*edem-2*::EDEM-2::mCherry transgenes. Exons are indicated by blue boxes separated by black lines indicating introns. The transgenes include the sequence of the first intron. **(C)** Quantification of CPL-1* fluorescence in *edem-1* and *edem-2* mutants harbouring or not EDEM-1 or EDEM-2 transgene. Dots in scatter gram represent mean fluorescence/μm^2^ x 1000). **(D)** EDEM-1::mCherry and EDEM-2::mCherry expressed from the transgenes localize correctly to ER. Confocal images of the ER marker GFP::SP12 and EDEM::mCherry in WT animals demonstrating ER colocalization in merged. Exposure adjustments were uniformly applied. **(E)** Survival fraction of the indicated strains upon 6h heat stress at 35°C. Each strain was scored on three replicate plates and each experiment was repeated independently four times. **(F)** Percentage of eggs that developed into L4 larvae after 3 days on 4 μg/ml tunicamycin. Each strain was scored in six independent experiments in triplicates. **(G)** Representative image of *edem-3* eggs that developed into adults after 10 days on tunicamycin (4 μg/ml). Presence of a mixt stage population and a grater number of eggs on *edem-3* plate at the time of observation indicate better survival on tunicamycin. The red bars in A and C indicate the mean ±SEM. Values inside the columns in E and F represent the total number of eggs analyzed. *P<0.05; **P<0.01; ***P<0.001; ****P<0.0001; ns, not significant.

Next, we examined the contribution of each EDEM to the clearance of misfolded CPL-1* from intestinal cells using the deletion alleles, which by contrast to RNAi silencing allows investigation of epistatic effects on CPL-1* clearance. We first tested the response of WT transgenic worms to increasing concentrations of kifunensine and we found that treatment with 30 μM kifunensine was sufficient to induce a maximal CPL-1* fluorescence in WT transgenic animals (S2A Fig). We used this treatment as a positive control to assess the effect of EDEM loss. Quantification of CPL-1* fluorescence showed increased fluorescence in both *edem-1* and *edem-2* mutants compared with WT control, and their effects were not synergistic nor additive (Fig 5A). This suggests that EDEM-1 and EDEM-2 rather work in a common degradation pathway to clear misfolded CPL-1* from the ER. *sel-1* mutants showed a higher fluorescence compared with WT, probably because SEL-1 works downstream of EDEM proteins in CPL-1* clearance and most likely is important for CPL-1* retrotranslocation. In contrast, *edem-3* mutants showed a CPL-1* fluorescence similar to WT control (Fig 5A). Also, *edem-3* loss decreased the fluorescence of both *edem-1;* CPL-1* and *edem-2*; CPL-1* single mutants, as well as *edem-1; edem-2;* CPL-1* double mutants (Fig 5A), indicating that *edem-3* loss has actually a beneficial effect on CPL-1* clearance. The simplest interpretation is that *edem-3* loss triggers activation of a mechanism that clears accumulation of CPL-1* in the intestine and this mechanism is partially dependent on functional EDEM-2 and EDEM-1. Treatment of WT animals with kifunensine showed similar fluorescence as for the *edem-TKO* triple mutant; CPL-1* mutants, indicating that there was no contribution to fluorescence from other ER mannosidases, i.e., ER manI.

EDEM-1::mCherry and EDEM-2::mCherry transgenes (Fig 5B) dramatically reduced the fluorescence level of CPL-1* in the corresponding mutant worms, as shown by both fluorescence quantifications and Western blotting (Figs 5C and S2B), indicating that the impairment of CPL-1* degradation is a consequence of *edem-1* and *edem-2* loss, respectively. Both EDEM-1::mCherry and EDEM-2::mCherry transgenes are correctly localized to ER in the intestinal cells, as denoted by the extensive overlapping with the ER marker GFP::SP12 [32] (Fig 5D). Interestingly, CPL-1* degradation in *edem-2* mutants was restored by EDEM-1 overexpression and vice versa, suggesting that overexpression of EDEM-1 and EDEM-2 could partially compensate for each other function under ER stress conditions (Fig 5C).

To determine whether the absence of EDEM proteins would affect the responsiveness to ER stress induced by other stimuli, we examined the sensitivity of *edem* mutants to the ER specific stressors tunicamycin and thapsigargin and to the heat stress, an ubiquitous folding stressor that affects all intracellular compartments. Exposure to 35° C for 6h decreased the survival rate of WT worms by more than 60% (Fig 5E). Whereas exposure to heat stress decreased survival of *edem-1* and *edem-3* single and double mutants as well as *sel-1* mutants similarly to WT controls, *edem-2* mutants showed improved survival, and this effect was further increased (to a small extent) by the addition of *edem-1* and *edem-3* mutations (Fig 5E). Pre-treatment of control animals with 15 μM kifunensine for 24h increased survival upon heat stress similarly to *edem-2* mutants suggesting that the improved survival is not a consequence of a linked background mutation but rather of a compromised ERAD. Overall, loss of EDEM-2 points towards a prominent role of this protein in the process that activates ER stress response that confers cellular protection against heat shock.

We also found that *edem-3* mutants as well as WT animals pretreated with kifunensine showed increased survival and development (versus WT controls) upon exposure to 4 μg/ml tunicamycin (Fig 5F), whereas WT, *edem-1*, *edem-2*, and *sel-1* mutants showed comparable sensitivity. The *edem-3* mutants that developed into adults survived a long time on plates with tunicamycin and produced viable progenies (Fig 5G). Addition of *edem-1* mutation had no significant effect on the development rate of *edem-3* mutants (Fig 5F), but addition of *edem-2* mutation slowed the development of *edem-3* mutants, although not statistically significant, suggesting that the improved development induced by *edem-3* loss might be partially dependent on functional EDEM-2. Again, under these ER stress conditions, EDEM-3 loss could trigger activation of a compensatory stress response to promote survival and growth. Notably, previous studies using cell culture showed that preconditioning with kifunensine reduced tunicamycin mediated cytotoxicity and increased cell viability [33]. Interestingly, only pre-treatment with kifunensine, which mimics chronic mild ER stress, rendered a protective effect and application of kifunensine at the start of tunicamycin treatment did not confer any protection [33]. The process of increasing stress resistance after a mild exposure to stress is an adaptive response known as hormesis [34]. We next assayed *edem* mutants for their sensitivity to thapsigargin, an inhibitor of ER calcium reuptake [35]. Since many ER chaperones require calcium for proper functioning, thapsigargin induces ER stress by impairing chaperone-assisted folding of misfolded proteins. In contrast to what we observed with the protein-folding inhibitor tunicamycin, thapsigargin failed to activate the expression of *edem* genes (S3A Fig), and moreover, all *edem* mutants as well as *sel-1* mutants showed comparable sensitivity with WT animals to 10 μM thapsigargin (S3B Fig).

Collectively, our findings indicate that the loss of EDEM functions impair clearance of misfolded proteins during prolonged/acute ER stress. Concomitantly, preconditioning to the mild ER stress induced by EDEM loss activates a hormetic response that confers cellular protection. It also suggests that EDEM relative contributions to these processes depend on the type and intensity of the ER stress the worms encounter, which correlates with *edem-1*, *edem-2* and *edem-3* individual expression patterns under physiological conditions and specific tissue activation upon ER stress (Fig 1C–1E).

### *C*. *elegans* EDEMs differentially influence lifespan

**Fig 6:**
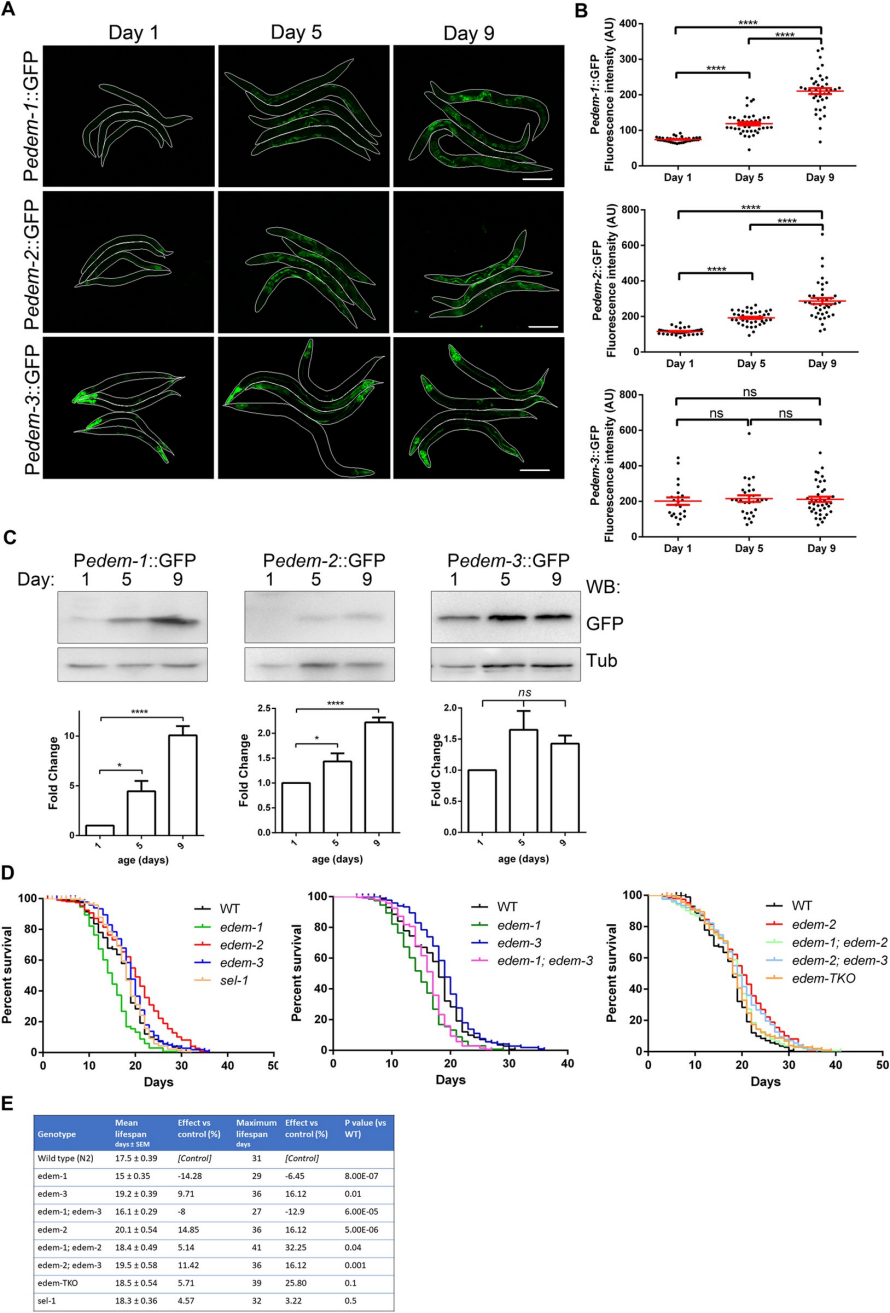
Aging-associated phenotypes of *edem* mutants under physiological conditions. **(A)** Representative images illustrating WT animals carrying P*edem*::GFP reporters at the indicated day of adulthood. The worms were outlined for better visualization. Exposure adjustments were uniformly applied. Scale bar 50 μm. **(B)** Fluorescence quantification of P*edem*::GFP transgenic animals presented in (A). Dots in scatter grams represent mean fluorescence in arbitrary units (AU) of P*edem*::GFP transgenic animals at the indicated ages. The red bars indicate the average ±SEM. ****P<0.0001; ns, not significant. **(C)** Immunoblot analysis of P*edem*::GFP transgenes expression during aging. Total protein lysates derived from WT animals carrying the P*edem*::GFP transgenes were separated by SDS-PAGE and immunoblotted with anti-GFP polyclonal antisera. Tubulin was used as loading control. The histograms show the densitometry values of P*edem*::GFP bands normalized to the value of day 1 (n = 4 ± SEM, one way ANOVA, Bonferroni’s multiple comparisons test), *P<0.05; ****P<0.0001; ns, not significant. **(D-E)** Lifespan analysis of WT and *edem* mutants. Graphs were plotted as Kaplan Meier survival curves of indicated *edem* mutants under normal conditions **(D)**. Lifespan values are summarized in **(E)**. *P* values were calculated by Mantel-Cox log-rank test. N = 70 animals per experiment in 3 independent experiments.

Decline of protein homeostasis is one of the major hallmarks of aging [36–38]. Activation of UPR^ER^ and, in general, initiation of protective mechanisms against ER stress decline with age, both in mammals and *C*. *elegans* [39,40]. Since activation of protective pathways that confer stress resistance often extends lifespan, we investigated the effect of *edem* deficiencies on lifespan under physiological conditions. First, we examined whether *edem* expressions change with age, hence, we monitored the temporal abundance of P*edem*::GFP transcriptional reporters in WT animals. We observed an increase in fluorescence level of all three P*edem*::GFP reporters between days 1, 5 and 9 of adulthood (Fig 6A). Expression of both P*edem-1*::GFP and P*edem-2*::GFP was mainly upregulated in intestinal cells, whereas P*edem-3*::GFP maintained expression in nervous system and pharynx, but increased the expression in posterior intestine, excretory channels and hypodermis, although there was variability in the penetrance of expression (Figs 6A and 1Ea). However, fluorescence quantification showed increased expression for P*edem-1*::GFP and P*edem-2*::GFP but not for P*edem-3*::GFP (Fig 6B). The auto-fluorescence gut granules increase with age and to exclude their contribution to the fluorescence in old animals we measured P*edem*::GFP expression levels by Western blotting. Between days 1, 5 and 9, P*edem-1*::GFP levels increased more than 4-fold, whereas P*edem-2*::GFP levels increased by almost 50% between day 1 and 5, and doubled between day 1 and 9 (Fig 6C). P*edem-3*::GFP showed a relatively constant level of expression between day 1, 5 and 9 (Fig 6C).

Next, to determine whether the increase of *edem* expression with age is protective or detrimental, we examined the effects of EDEM loss on *C*. *elegans* lifespan under physiological conditions. We observed a 14% reduction in the mean lifespan and a 6% reduction in the maximum lifespan for *edem-1* mutants relative to WT animals, (Fig 6D and 6E), whereas *edem-2* mutants showed 14% and 16% increase in mean and maximum lifespan, respectively, relative to WT animals (Fig 6D and 6E). However, since *edem-2* mutants also showed retarded development, the lifespan extension could reflect their slow growth. The *edem-3* mutants showed a modest increase in mean and maximum lifespan (9% and 16%, respectively). Addition of the *edem-1* and/or *edem-3* mutation to *edem-2* mutants slightly shortened the mean lifespan of *edem-2* mutants comparative to WT animals (Fig 6E). These results suggest that *edem-2* and *edem-3* might negatively regulate lifespan, whereas increasing levels of *edem-1* expression in old age might act as a mechanism aiming to maintain normal lifespan.

### EDEM deficiency induces a weak sensitization of UPR^ER^

**Fig 7:**
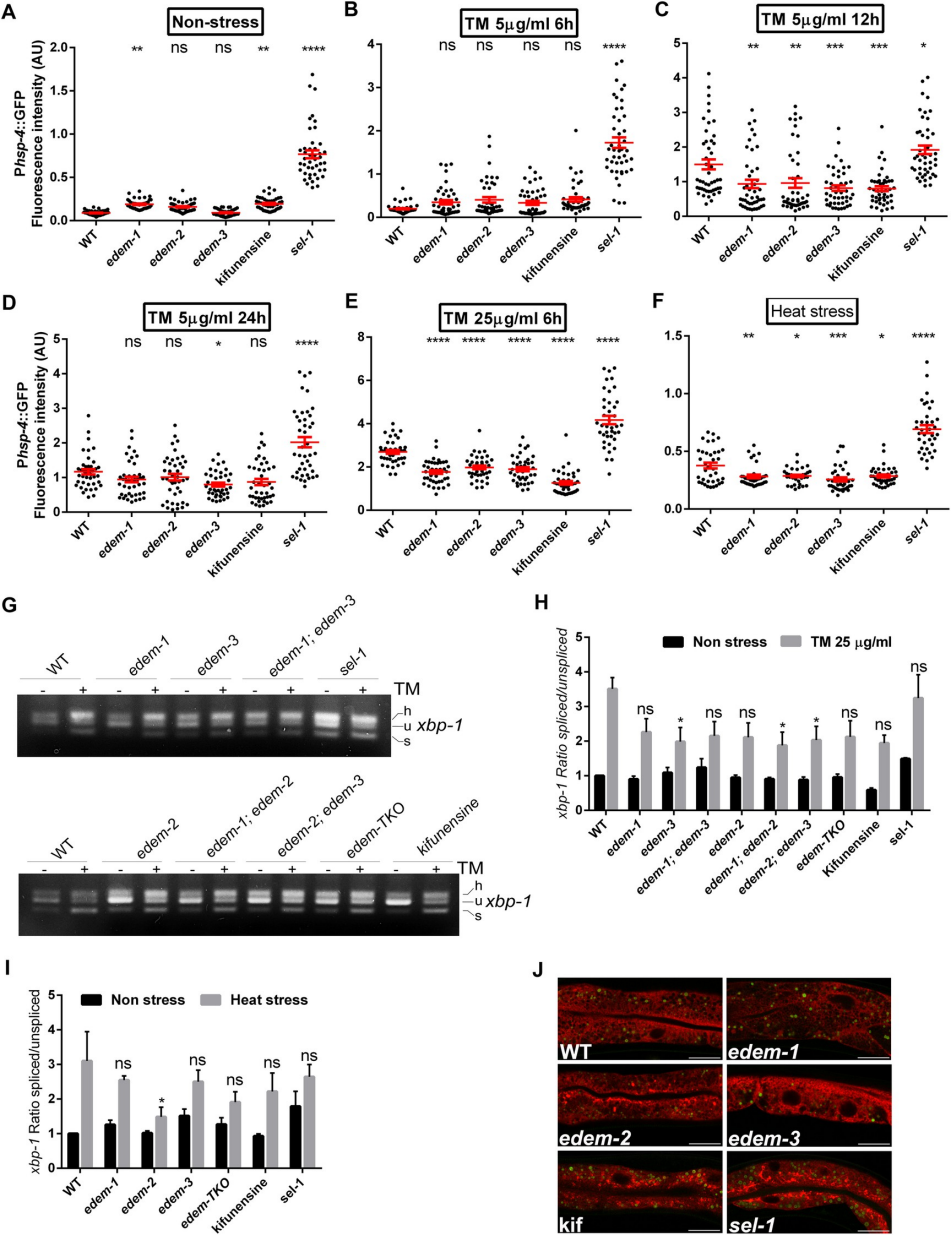
EDEM depletion mitigates the ER stress without activation of IRE-1/XBP-1 pathway. **(A-F)** Quantification of WT (EV) or RNAi-treated day one adults carrying P*hsp-4*::GFP transgene, subjected or not to the indicated tunicamycin and heat shock treatment. The worms were kept on RNAi plates for one generation before exposure to ER stress treatments. Values represent mean fluorescence/μm^2^ x 1000. The red bars indicate the average ±SEM **(G-H)** Young adults were non-treated (NS) or treated with tunicamycin (25 μg/ml, 6h) for *xbp-1* splicing from indicated strains. Total RNA was isolated, reverse transcribed and used for PCR analysis of *xbp-1* splicing forms. In **(G)** h-hybrid, u-unspliced and s-spliced denote the forms of *xbp-1* mRNA detected in the agarose gel. **(H)** Band intensity of samples was quantified and the ratios of spliced/unspliced *xbp-1* normalized to WT untreated samples were plotted in the graph. Values in the graph represent the mean of 4 independent experiments ± SEM (Two way ANOVA, Dunnett’s multiple comparisons test). **(I)** Young adults were non-treated (NS) or heat shocked (4h at 35°C) for *xbp-1* splicing from indicated strains. Total RNA was isolated, reverse transcribed and used for PCR analysis of *xbp-1* splicing forms. (Two way ANOVA, Dunnett’s multiple comparisons test). **(J)** Confocal images of SEL-1(1–79)::mCherry::HDEL intestines of indicated strains grown under physiological conditions. In green are indicated the autofluorescent gut granules captured with the Argon laser. Both channels were set on the same track. The images are representative for the phenotypes seen in more than 60% of the mutants. Exposure adjustments were uniformly applied. Scale bar 20μm. *P<0.05; **P<0.01 ***P<0.001; ****P<0.0001; ns, not significant.

In eukaryotes, the canonical UPR^ER^ is activated upon ER stress to ameliorate accumulation of defective proteins in the ER and to re-establish ER homeostasis [27–29].[We next investigated whether the increased resistance to ER stress conferred by the loss of EDEM-2 and EDEM-3 could reflect UPR^ER^ activation. Transcription of *hsp-4*, the *C*. *elegans* orthologue of mammalian GRP78/BiP chaperone, is elevated in response to ER accumulation of misfolded proteins [29,30]. As such, quantification of P*hsp-4*::GFP fluorescence is widely used as a quantitative readout of UPR^ER^ activation. Disruption of ERAD by RNAi-mediated downregulation of *edem-1* increased the basal expression of P*hsp-4*::GFP reporter, but to a 4x lower extent than *sel-1* RNAi, whereas downregulation of *edem-2* and *edem-3* by RNAi did not result in a statistically significant change compared to WT (EV) control (Figs 7A and S4A). Treatment of WT (EV) control worms with kifunensine increased the basal expression of P*hsp-4*::GFP to the same extent as *edem-1* RNAi treatments, confirming that ERAD impairment through inhibition of mannosidase activity induces only a weak sensitization of UPR^ER^ under physiological conditions. In all cases, the increase of P*hsp-4*::GFP fluorescence was mainly seen in intestine and hypodermis (S4A Fig).

We then assessed the activation of UPR^ER^ upon ER stress in worms treated with *edem* RNAi. ER stress induced by exposure to lower dose (5 μg/ml for 6h, 12h and 24h) as well as high dose (25 μg/ml, 6h) of tunicamycin increased P*hsp-4*::GFP expression in WT (EV) control worms (Figs 7B–7E and S4A). Short exposure to low dose (5 μg/ml for 6h) of tunicamycin did not significantly affect P*hsp-4*::GFP expression in *edem* RNAi-treated animals or in WT worms treated with kifunensine, when compare with WT control worms (Fig 7B). In contrast, longer exposure to low dose (5 μg/ml for 12h and 24h) or short exposure to high dose (25 μg/ml for 6h) of tunicamycin decreased the expression of P*hsp-4*::GFP in all *edem* RNAi treated worms, when compare with WT control worms (Fig 7C–7E). The reduced expression of P*hsp-4*::GFP upon tunicamycin treatments induced by *edem* downregulation or kifunensine treatments was the most prominent in the intestine (S4A Fig). This response was found to be *edem* specific since disruption of ERAD by s*el-1* RNAi downregulation increased P*hsp-4*::GFP expression upon tunicamycin treatments, regardless of the time or dose exposure (Figs 7B–7E and S4A). Similar effects were observed when heat shock was used to induce ER stress. The increased P*hsp-4*::GFP expression in WT(EV) animals induced by heat shock was attenuated by *edem* RNAi downregulation as well as kifunensine pre-treatment but not by *sel-1* downregulation (Fig 7F). In contrast, upon exposure to 5 μM thapsigargin, the P*hsp-4*::GFP expression in *edem-1* and *edem-2* RNAi-treated animals was similar to that of WT animals, but increased in *edem-3* and *sel-1* RNAi-treated animals (S3C Fig). All these results suggest that EDEM deficiencies alter the ER stress signalling transduction and implicitly the activity of UPR^ER^.

We then investigated the correlation of P*hsp-4*::GFP expression with the activity of IRE-1 by monitoring *xbp-1* mRNA splicing in *edem* mutants both in basal conditions and upon ER stress. Tunicamycin treatment increased the basal level of *xbp-1s* mRNA spliced form by more than 3x (Fig 7G and 7H). This response was attenuated in all *edem* mutants, although only in *edem-3*, *edem-1; edem-2* and *edem-2; edem-3* mutants the differences were statistically significant (Fig 7G and 7H). We also found that the loss of EDEM-1 and EDEM-3 had no significant effect on *xbp-1s* expression upon heat stress, but loss of EDEM-2 decreased *xbp-1s* expression (Fig 7I). In contrast, loss of EDEM did not significantly affect *xbp-1’s* level upon thapsigargin treatment (S3D Fig). Taken together, down regulation of *xbp-1s* and *hsp-4* levels by mutations in *edem* genes suggest that preconditioning to chronic loss of EDEM is accompanied by a decrease in ER stress, which depends on the type and intensity of the ER stressor.

ERAD impairment by genetic inactivation of *edem-1* and *edem-3* did not dramatically alter the ER morphology in intestinal cells under non-ER stress, as revealed by the ER marker SEL-1(1–79)::mCherry::HDEL (Fig 7J). In *edem-2*, *sel-1* and animals treated with kifunensine, the ER marker showed disturbed ER morphology, including lower abundance of ER tubules and expansion of the existing ones, especially toward the apical membrane of intestinal cells. In addition, bright circular structures derived probably from fragmentation of ER tubules were observed in *edem-2* and *sel-1* mutants as well as worms treated with kifunensine. Disturbance of the ER network has also been observed in *C*. *elegans* Derlin deficient animals, where the perinuclear concentration of ER observed in wild type coelomocytes was lost and the ER exhibited expanded tubules spread all-over the cell [41]. Given the defective activation of the IRE-1 branch of canonical UPR^ER^ that we observed upon the loss of EDEM activity but not SEL-1, corroborated with improved survival of *edem* mutants under ER stress conditions, it is likely that ER-preconditioning/ER-hormesis induced by EDEM-dependent impairment of ERAD activates an adaptive ER stress response to restore homeostasis and to protect the worms against subsequent insults.

### EDEM response to ER stress modulates UPR activity

**Fig 8:**
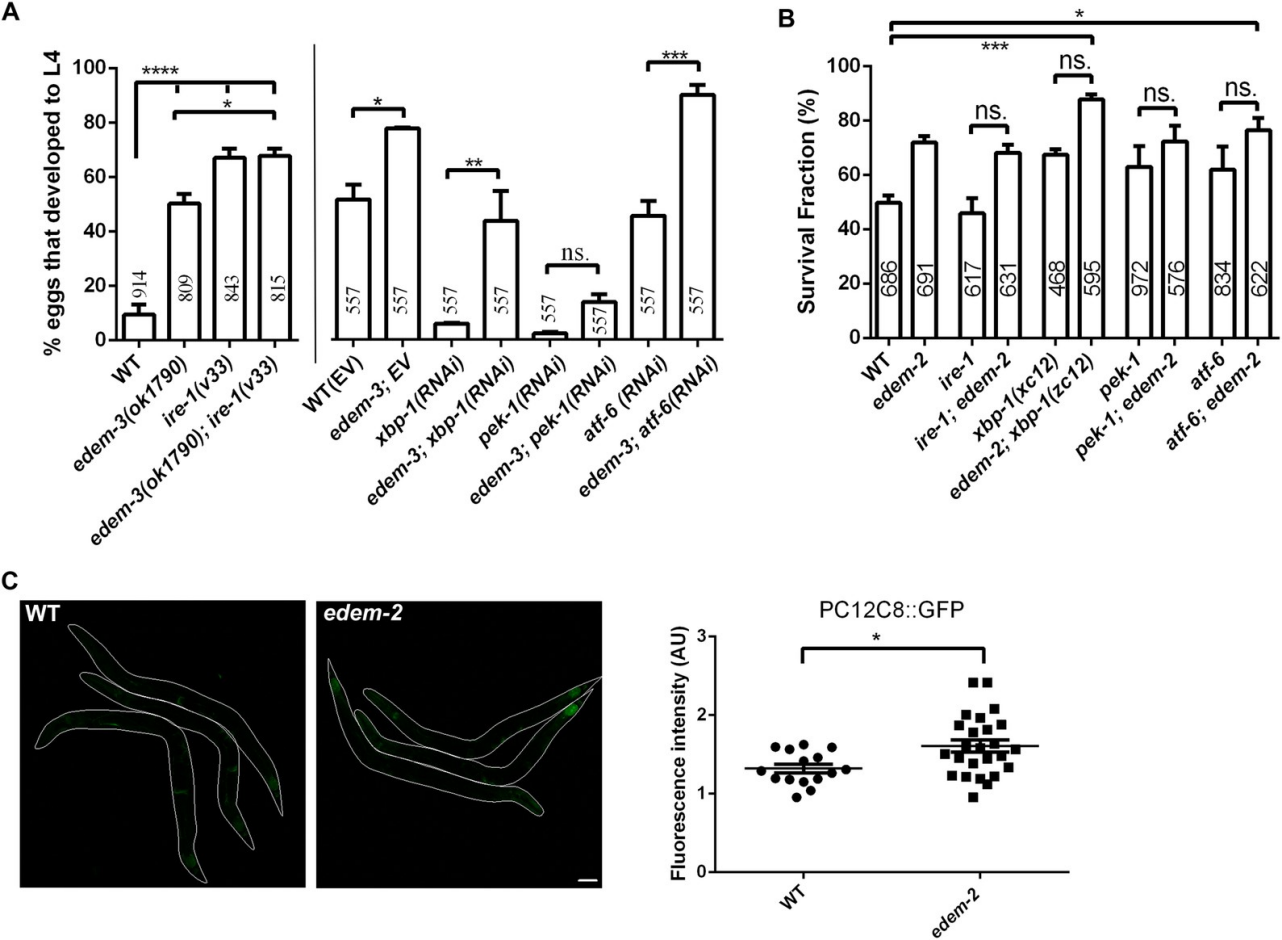
Loss of EDEM-2 and EDEM-3 induces a similar ER stress response as IRE-1 and XBP-1 deficient animals. **(A)** Percentage of eggs that developed into L4 larvae after 3 days on 4 μg/ml tunicamycin. Each strain was scored in more than three independent experiments in triplicates. **(B)** Survival fraction of the indicated strains upon 4h heat stress at 35°C. Each strain was scored on three replicate plates and each experiment was repeated independently three times. **(C)** Representative confocal images of WT control or *edem-2* young adults carrying the *hsp-70*::*GFP* (P*C12C8*.*1*::GFP) transgene under heat shock treatment (30 minutes at 35°C, with 3h recovery). For *edem-2* mutants, the strongest expression was observed in the intestine. The worms were outlined for better visualization. Images were obtained using the same confocal settings. Exposure adjustments were uniformly applied. Scale bar: 50 μm. **(D)** Fluorescence quantification of P*hsp-70* (P*C12C8*.*1*::GFP) transgenic animals presented in **(C)**. Dots in scatter grams represent mean fluorescence in arbitrary units (AU). The bars indicate the average ±SEM. *P<0.05.

Due to the central role of UPR^ER^ in response to ER stress we tested whether activation of canonical UPR^ER^ plays a role in the attenuation of ER stress induced by *edem-3* and *edem-2* loss. We constructed *edem-3* double mutants with the null allele of *ire-1(v33)* or fed *edem-3* mutants dsRNA to inhibit *xbp-1*, *pek-1* (the worm orthologue of mammalian PERK) and *atf-6*, respectively. The *ire-1(v33)* mutants showed increased growth upon tunicamycin stress (Fig 8A). The growth rate of *ire-1; edem-3* double mutants was similar to that of *ire-1* mutants (Fig 8A), indicating activation of a common mechanism that confers protection against tunicamycin stress. Down regulation of *xbp-1* and *pek-1* but not *atf-6* by RNAi dramatically impaired the growth of *edem-3* mutants upon tunicamycin treatment (Fig 8A). Thus, *edem-3* regulates the growth rate under tunicamycin stress mainly through *pek-1* pathway and partially through *xbp-1* pathway.

We also constructed *edem-2* double mutants with null alleles of *ire-1(v33)*, *xbp-1(zc12)*, *pek-1(ok275)* and *atf-6(ok551)* and monitored the survival rate upon heat stress. Upon 4h heat stress at 35° C, the *ire-1(v33)*, *xbp-1(zc12)*, *pek-1(ok275)* and *atf-6(ok551)* single mutants showed decreased survival similarly to WT controls (Fig 8B). Addition of *edem-2* mutation significantly increased the survival rate of *xbp-1* and *atf-6* mutants, when compared with WT control (Fig 8B). However, addition of *edem-2* mutation did not have an additive or synergistic effect on survival rate of any UPR^ER^ mutants, suggesting that decreased activity of UPR^ER^ has an important role in conferring protection against proteotoxic stress. Notably, in cell culture, cells continuously exposed to prolonged ER stress attenuate activation of UPR to facilitate adaptation [42,43]. Because the heat shock activates expression of many genes, including cytoplasmic chaperones, we next monitored the expression of the major heat-inducible cytoplasmic *hsp70*, C12C8.1 [44] both in WT controls and *edem-2* mutants. Under non stress conditions, *hsp70* (PC12C8.1::GFP) is not expressed in *edem-2* mutants (S4D Fig). Exposure to a short increase in temperature (35° C for 30 min) activated the *hsp70* reporter in many tissues, both in WT controls and *edem-2* mutants (Fig 8C). Fluorescence quantification showed a slightly but significantly increased expression of *hsp70* reporter in *edem-2* mutants (Fig 8D), especially in intestinal cells when compared with WT animals.

We then quantified by qPCR the *hsp-4* mRNA level in *edem* mutants upon tunicamycin and heat shock treatment, respectively, to find if the decrease in P*hsp-4*::GFP protein fluorescence is also apparent at the mRNA level. There were no significant differences between the levels of *hsp-4* mRNA in *edem* mutants and WT animals (S4B and S4C Fig), suggesting that attenuation of GFP translation contributed to the lower P*hsp-4*::GFP fluorescence levels (Fig 7E and 7F) in these animals.

Altogether, our results show that preconditioning to mild ER stress induced by EDEM loss generates an integrated ER stress response that depends at least partially on the activity of the UPR sensors. This response could overpass intercellular compartments boundaries to enhance proteostasis capacity and to protect the worms against subsequent insults.

## Discussion

We report here the first characterization of combined roles for *edem-1*, *edem-2* and *edem-3* functions under physiological and ER stress conditions in a model organism that possesses three individual *edem* genes, as humans. Yeasts have only one member of ER α-mannosidase family proteins, Htm1p, which performs the quality control in the ER [45], whereas the fruit fly has only two EDEM proteins responsible for the protein quality control in ER [46]. As in mammals, *C*. *elegans* has three EDEM proteins, which we show to perform an evolutionary conserved function in mediating degradation of misfolded polypeptides and reducing ER stress. We find that EDEM-2 has a major role in ERAD under physiological conditions, whereas EDEM-1 and EDEM-3 roles become prominent under ER stress conditions. To maintain ER homeostasis under ER stress conditions the UPR^ER^ is activated, both in mammals and *C*. *elegans* [47]. However, we found that EDEM-2 and EDEM-3 proteins are important factors in modulating ER stress responsiveness. Thus, opposite to *sel-1* mutants, the *edem* mutants exhibited reduced basal expression of both *xbp-1s* and GRP78/BiP homologue, *hsp-4*, and mitigated their expression upon ER stress, in association with increased resistance and survival under ER stress conditions. These observations do not suggest a merely decrease of sensitivity to ER stress, but activation of a hormetic protection triggered by the fine-tuning response to ER stress under EDEM loss pre-conditioning. Notably, the effect of EDEM loss on ER stress resistance and survival under stress was similar to that exerted by the ERAD inhibitor, kifunensine. This further validates a specific function of EDEM in the ERAD processes in *C*.*elegans*.

Understanding how distinct pathways modulate ER homeostasis at the organism level under physiological and stress conditions is a critical question mark in elucidating the pathogenesis of many human diseases. Cells respond to ER stress in two consecutive phases that involve different effectors. A subtoxic level of ER stress maintains in a first phase a moderate level of UPR activity that stimulates adaptive defences to promote cell survival by activating cytoprotective mechanisms. When adaptive mechanisms fail to compensate for an excessive ER stress, the UPR is activated in an intense and sustained manner, leading to a cytotoxic response that stimulates cell death [43,48]. Rather than harmful, preconditioning to mild ER stress either by pharmacologic or genetic inactivation has been shown to activate a hormetic response that confers protection against subsequent toxic insults [43,49]. For example, mild ER stress preconditioning or IRE1 deficiency induces a protective response in animal and cellular models of neurodegenerative diseases by activating autophagy [50–53]. In *C*. *elegans*, animals with dysfunctional UPR^ER^ and ERAD are viable under physiological conditions but show reduced survival and delayed development upon ER stress [27,54,55]. We found that in contrast to SEL-1L loss, impairment of ERAD by EDEM loss or pretreatment with kifunensine triggers only a mild ER stress in *C*. *elegans*, which further enhances responsiveness to acute ER stress to promote survival, thus revealing a novel role for EDEM in fine-tuning the ER stress responsiveness.

Expression of *C*. *elegans edem* genes is observed in specific cells and tissues. *edem-1* and *edem-2* are constitutively expressed in cells with high secretory activity as intestinal cells, hindgut and pm6 cells (responsible for chitin synthesis and secretion). Instead, *edem-3* has a distinct expression pattern, being mainly expressed in the pharynx and nervous system. However, consistent with a role for EDEMs in ERAD, their expression can be upregulated in other tissues upon ER stress. Taking into consideration that double and triple mutants do not show additive or synergistic effects it is possible that EDEM proteins work together for degradation of misfolded proteins, all three being ubiquitously expressed under physiological conditions but in some tissues their expression may be below the level of detection.

In mammals, misfolded polypeptides from ER are transported into specialized ER quality control compartments (ERQC), where terminally misfolded polypeptides are recognized and targeted for ERAD. ERmanI was found confined with the ERAD substrates in ERQC compartments [20,53], extracting misfolded polypeptides from the calnexin cycle, and thus accelerating their degradation via ERAD [56,57]. Therefore, it has been postulated that ERmanI has a central role in the first step of ERAD in higher organisms, as it has been described in yeast [58]. However, this hypothesis has been challenged by other reports that showed that knockout of ERmanI in mammalian cell culture has a low effect in ERAD initiation, and EDEM2 initiates ERAD by trimming the first mannose residue [18,59,60], whereas EDEM3 and EDEM1 remove additional mannose residues [18]. Similar to vertebrates, most of the *C*. *elegans* N-glycans are high mannose type (Man5-9GlcNAc2), although they have distinct mannose derivatives, whereas complex and hybrid N-glycans are absent or poorly represented [61,62]. Our phenotype and epistasis analyses indicate that the *C*. *elegans* EDEM-2 is the core component of a conserved mechanism of protein quality control in ER and its activity is critical for engagement of terminally misfolded proteins to the ERAD pathway under physiological conditions. This is further supported by the recent identification of a number of endogenous glycoproteins whose ERAD is EDEM2 dependent in mammals [63]. In *C*. *elegans*, EDEM-2 loss not only confers phenotypic pleiotropy under physiological conditions, but *edem-2* mutants show the most severe phenotypic alterations that are not phenocopied by *edem-1* and *edem-3* single or *edem-1; edem-3* double mutants. Moreover, neither *edem-1* nor *edem-3* mutations dramatically alter the morphological or physiological defects of *edem-2* mutants under non-stress conditions, suggesting that all three EDEM may act in a common degradation pathway, with EDEM-2 acting as a major player. Although we did not specifically address the role of ERmanI in this work, we treated WT worms with kifunensine, which inhibits all ER mannosidases including ERmanI [64,65], to compare with the effect of EDEM loss. WT animals treated with kifunensine responded constantly in a similar manner as *edem* mutants in all tests, which suggests that the *C*. *elegans* ERmanI might also have a minor role in the quality control of misfolded proteins, as it was suggested by knock-out experiments in cell culture.

Our data argue that upon inducing ER stress, the role of EDEM-1 and EDEM-3 for the processing of misfolded proteins becomes prominent. The differences in the ER stress response of *edem* mutants that we observed in our tests could imply that EDEM proteins process different ERAD substrates or have cell-dependent interactors that could trigger different outcome effects as it was shown in mammalian systems [19,66,67]. We observe that *edem* mutants show different behaviour to the treatment with tunicamycin and thapsigargin (resistance to tunicamycin, sensitivity to thapsigargin), although both are ER specific folding stressors. This could be because of their different kinetics and mechanism of action: tunicamycin induces protein misfolding in a slow manner since it depends on protein biosynthesis to exert its action, whereas thapsigargin disrupts protein folding in a rapid and robust manner leading to a quick UPR response. It has been reported that overexpression of neuronal *xbp-1s* increases the stress resistance and lifespan of *C*. *elegans* in a cell non-autonomous manner [40]. We cannot exclude the possibility that stress resistance induced by EDEM-3 loss is not a consequence of neuronal modulation of *xbp-1s* expression, since EDEM-3 is mainly expressed in the nervous system. However, downregulation of *edem-3* by RNAi, which is not effective in the nervous system, mitigated the P*hsp-4*::GFP expression in the intestine (S4 Fig), and moreover, misfolded CPL-1* did not accumulate in the intestine of animals fed with *edem-3* RNAi (Fig 2A). Thus, it seems likely that the response to intestinal ER stress in the absence of *edem-3* is regulated through a cell autonomous mechanism whereby a low level of IRE-1/XBP-1 activation and implicitly ERAD is needed to restore ER homeostasis. This hypothesis is consistent with the existence of a dynamic auto-regulatory circuit between IRE-1α/XBP-1 and ERAD activity, whereby ERAD restrains IRE-1 activity by promoting its degradation [68] and IRE-1 activation promotes ERAD. Conversely, in WT animals with intact EDEM, a potential ER stress feed forward loop could occur, wherein XBP-1s might upregulate *edem* expression in response to ER stress, and in turn, *edem* upregulation could promote XBP-1 dependent activation of UPR^ER^. Such positive feed forward loop could be required in WT animals for an enforced response to acute ER stress to maintain ER homeostasis.

Whilst ERAD have been clearly implicated in relieving the ER stress upon XBP-1s induction, there is limited information directly linking ERAD to stress resistance. Thus, how ERAD impairment could induce stress resistance? Recent evidence shows that in addition to its canonical function in degradation of misfolded polypeptides, ERAD is also involved in regulation of gene expression. One example is p97/CDC-48 that has been shown to modulate expression of ER stress response genes by degradation of RUVB-2/Reptin transcriptional repressor, both in *C*. *elegans* and human cells [69]. Under non stress conditions the RUVB-2/Reptin suppresses expression of a set of ER-stress-associated UPR^ER^ genes. Upon ER stress, RUVB-2/Reptin is degraded in a p97/CDC-48-dependant manner, thus relieving repression of the UPR^ER^ genes. Conversely, in the absence of p97/CDC-48 the RUVB-2/Reptin is not degraded anymore and the UPR^ER^ is maintained at basal level even under ER stress conditions [68].

In conclusion, we show that EDEMs respond differently to ER stress stimuli, and moreover, EDEMs deficiencies activate an adaptive program to promote organism survival under acute ER stress. In this context, given the evolutionary conservation of ERAD machinery in *C*. *elegans*, our findings underlie a new conceptual model for regulation of ER homeostasis and raise the possibility that modulation of ERAD activity could be employed for therapeutic interventions to mitigate ER stress toxicity associated with human diseases.

## Materials and methods

### Strains and genetic methods

*C*. *elegans* strains were cultivated at 20°C, otherwise indicated and fed with *E*. *coli* OP50. The strains used in this study are listed in S1 Table. The *edem-2(tm5186)* strain was outcrossed 12 times, whereas *edem-1(tm5068)*, *edem-3(ok1790)* and *sel-1(tm3901)* 8 times. The primers for genotyping are listed in S2 Table.

The *edem-1; edem-2* and *edem-2; edem-3* double mutants were obtained with the help of *mIs10*::GFP balancer. The *edem-TKO* triple mutants were obtained by crossing *edem-1; +/mIs10*::GFP; ^*o*^*/edem-3* males to *edem-2; edem-3* hermaphrodites. Single *edem-1* and *edem-2* mutant worms bearing fluorescent transgenes were constructed with the help of *mIs11*::GFP and *mIs10*::GFP balancers, respectively. To obtain *edem-3*; transgenic mutants, transgenic males were crossed to *edem-3* hermaphrodites, then backcrossed to *edem-3*. The triple mutants bearing CPL-1* fluorescent transgenes were obtained by crossing *edem-1; +/mIs10*::GFP; ^*o*^*/edem-3* males to *edem-2; edem-3;* CPL-1* hermaphrodites. All the newly constructed strains were genotyped by PCR to ensure the presence of homozygous *edem* mutation alleles.

RNAi feeding assay was carried out as previously reported [70]. The *edem-1*, *edem-2*, and *edem-3* RNAi clones were constructed by cloning the full cDNA into L4440 vector and transforming HT115 bacteria. The *sel-1*, *xbp-1*, *pek-1 and atf-6* RNAi clones were obtained from Ahringer library. The efficiency of Ahringer RNAi clones was verified by monitoring their effect on P*hsp-4*::GFP reporter. The WT (EV) control for the RNAi experiments was the empty vector L4440.

### Molecular methods and transgenic animals

To produce P*edem-1*::GFP, P*edem-2*::GFP, P*edem-3*::GFP constructs, about 2.2 kb DNA sequence located immediately upstream of each *edem* was cloned into the pPD95.75 vector, respectively. To obtain translational reporter constructs for *edem-1* and *edem-2*, a genomic DNA sequence consisting of about 2.2kb of the promoter, first exon, first intron, and the rest of cDNA was fused in frame to mCherry or GFP, respectively. The translational reporter for *edem-3* was constructed in a similar way. To obtain transgenic animals, in each case 100 ng/ml of the transgene was co-injected with pRF4 vector (50 ng/ml) that expresses the *rol-6(su1006)* dominant marker. Transgenic P*edem-3*::EDEM-3::GFP animals did not produce any stable line, therefore *edem-3* expression pattern was established using transcriptional reporters. We found a similar expression pattern between transcriptional and translational constructs, for both *edem-1* and *edem-2* genes.

### Drug treatment and quantitative real-time RT-PCR

Synchronized first day young adult worms were kept on plates with 5 or 25 μg/ml tunicamycin (sc-3506, Santa Cruz Biotechnology, Heidelberg, Germany) for 6h and worms were immediately collected for RNA extraction. For thapsigargin (sc-24017, Santa Cruz Biotechnology, Heidelberg, Germany), treatment, synchronized first day adult worms were kept on plates with 5 μM thapsigargin for 4h and collected for RNA extraction. For heat stress treatment, synchronized WT first day young adult worms were kept for 4h at 37°C, then collected for RNA extraction. For osmotic stress treatments, L4 larvae were kept on plates with 200 mM NaCl for 24h, then collected for RNA extraction. Total RNA was extracted with Trizol reagent (15596018, Thermo Fisher Scientific) followed by retention on RNeasy columns (Qiagen). Total RNA (0.2 μg) was reverse transcribed into cDNA using First Strand cDNA kit (Thermo Fisher Scientific). Specific transcript levels were determined using QuantStudio 7 real-time PCR machine (Applied Biosystems) and SensiFast cDNA synthesis kit (Bioline). Most of the primer pairs were designed with the help of Primer-Blast software (NIH) and chosen to span one exon/exon junction (listed in S2 Table). Five reference genes (*cdc-42*, Y47F10D.4, *pmp-3*, *ama-1*, and *tbp-1*) were tested for stability of expression, and *pmp-3* and dcd-42 mRNA level was found to be the most stable under tunicamycin, thapsigargin and heat shock treatment. The transcript levels were normalized to *pmp-3* and cdc-42 levels. Fold changes were obtained by comparing the mRNA level exposed to ER stress conditions with transcript levels from WT non-stressed animals. At least three independent assays were performed for each experiment.

### Growth, lethality and brood assays

To score growth, 3–7 hermaphrodites were let to lay eggs for 2-3h, the eggs on each plate were scored, then 52h (for experiments conducted at 20°C) or 48h (for experiments conducted at 25°C) later the hatchlings that reached L4 stage were counted. For analysis of lethality and brood size, L4 worms were placed onto NGM plates seeded with OP50 and transferred every 12h to fresh plates until they ceased laying eggs. The dead eggs were scored about 30 h after the parent was removed from the plate and dead or arrested larvae were scored 3 days later. Parent worms that died or ran away from the plates were censored. To score growth, lethality and brood at 25°C, worms were kept at 25°C for one generation before analysis. To score growth upon tunicamycin-stress and thapsigargin-stress, 3–7 hermaphrodites were let to lay eggs for about 2–3 h on plates containing either tunicamycin (2 or 4 μg/ml), 10 μM thapsigargin or DMSO, and 3 days later the hatchlings that reached L4 stage were counted. To score survival upon heat stress, synchronized L4 larvae were kept for 6h at 35°C, returned to 20°C and 24h later the percentage of animals that were moving or responded to wire touch was scored. To analyze worms treated with kifunensine, the worms were pre-treated with 30 μM kifunensine for 24 h then heat shocked, whereas in the case of tunicamycin treatments the parent worms were pre-treated with 15 μM kifunensine, then let to lay eggs on plates with 30 μM kifunensine and indicated concentration of tunicamycin. The slow-growing strains were let to lay eggs with 8-10h in advance to obtain the synchronized strains subjected to tunicamycin or heat stress.

### Lifespan determination

For lifespan experiments, L4 larvae were manually selected at the final stage of vulva maturation. The worms were transferred to fresh plate every day until they cease laying eggs and then every 2–4 days; dead or alive animals were scored every day. Worms that crawled up the side of the dish, exhibited externalization of internal organs or internal hatching were censored. Lifespan assays were conducted at 20°C and included 3 independent experiments with 70 worms per experiment. In each experiment, all strains were assessed in parallel. Kaplan-Meier survival curves and Mantel-Cox log-rank tests were performed using the R program. Figures display Kaplan Meier survival curves of the pooled populations.

### Lysates preparation and immunoblot analysis

150 day 1, day 5 and day 9 adult worms were collected in PBS completed with 0.2% Triton X100, snap frozen in liquid nitrogen and stored at -80°C. For CPL-1* immunoblot 150 day 1 adult worms were collected in PBS completed with 1% TritonX100 and 1% NP40. The samples were thawed on ice, mixed with Laemmli-buffer containing 5% beta mercaptoethanol, heated to 95°C for 10 min and centrifuged at 16,000g for 5 min to pellet debris. Lysates were run on acrylamide gel and transferred onto nitrocellulose membrane. The membrane was incubated with anti-GFP antibody (ab290, Abcam) in a 1:5000 dilution in 3% BSA overnight at 4°C. Next day the membrane was incubated with HRP-coupled donkey-anti rabbit antibodies (Jackson Immunoresearch), applied in 1:20000 dilution in TBS with 0.1% Tween 20 for 1h. Signals were detected using ECL Western blotting solution and ECL Hyperfilm (GE Healthcare). Mouse anti-tubulin (T6199, Sigma) antibodies were used as loading control in the same conditions as above.

### Microscopy

Images were acquired using a Zeiss LSM 710 laser scanning confocal microscope with a pinhole of 1.0 Airy Units and band-pass filters such that there was no overlap between channels, when more than one channel was used for acquisition. Images were acquired with identical confocal settings for each reporter. The worms were anesthetized in 1mM levamisole and placed on a 2% agarose pad. The lipophilic dye DiI (Molecular Probes, V22889) was used to identify a subset of sensory neurons expressing P*edem-3*::GFP reporter. DiI specifically stains six amphid neurons in *C*. *elegans*: ASK, ADL, ASI, AWB, ASH, and ASJ. Well fed young animals were incubated for 2 h at room temperature with 200 μl of M9 containing 1::200 dilution of DiI, washed 3x with M9, and let to crawl for 1–2 h to remove excess dye before visualization. For the FM4-64 stained embryos the gravid worms were cut in egg buffer containing 2 μg/ml FM4-64 and immediately imaged. For the analysis of intestinal ER marker SEL-1(1–79)::mCherry::HDEL, the mCherry signals were detected at 500–520 nm and images taken in the GFP channel were used to identify the autofluorescence of gut granules. Figures were constructed with Photoshop CS5 and representative images are shown.

### Fluorescence quantifications

For quantification measurements the images were acquired with Zeiss LSM 710 confocal microscope using 10x objective and identical acquisition settings for each marker. For P*hsp-4*::GFP quantifications upon heat stress, the worms were heated for 4h at 35°C, immediately collected in 2% formaldehyde and kept on ice until visualization. Young adults around the stage of first ovulation were photographed, outlined, and selected as region of interest (ROI) to determine the mean fluorescence intensity and area using the measurement features of the Zen 2010 software.

### Statistics

All experiments were repeated independently at least three times. GraphPad Prism 6 software package was used for statistical analyses. Data are given as mean ± SEM and differences among groups were determined by one way ANOVA with Tukey’s multiple comparisons test if not otherwise indicated. The log-rank (Mantel-Cox) test was used to evaluate differences between survivals and to determine *P* values in lifespan assays.

## Supporting information

S1 Fig Immunoblot analysis of P*edem*::GFP expression in young animals. **(A)** Quantitative real-time PCR (qPCR) of *edem-3* mRNA. WT and *edem-3* mutant worms treated with empty vector or *edem-3* RNAi; expression were normalized to that of *cdc-42* and *pmp-3*. **(B)** Total protein lysates derived from WT P*edem*::GFP transgenic animals subjected to the indicated treatment were separated by SDS-PAGE and immunoblotted with anti-GFP polyclonal antisera; tubulin was used as loading control. NS- non-treated, TM- tunicamycin, HS-heat stress, OS- osmotic stress. The histograms show the densitometry values of P*edem*::GFP bands normalized to the value of of NS condition (n = 3 ± SEM, *t* test), *P<0.05; **P<0.01; ***P<0.001; *ns*, not significant. **(C)** RNAi downregulation of *edem* triggered accumulation of CPL-1* in intestinal cells. To overrule a significant contribution of autofluorescent stress granules to the GFP fluorescence, images captured with Diode laser were included. Scale bar: 20 μm.
(DOCX)

S2 Fig EDEM-1 and EDEM-2 are required for CPL-1* degradation. **(A)** Fluorescence intensities of WT animals carrying the CPL-1* transgene treated with indicated concentrations of kifunensine. Values in scatter gram represent mean fluorescence/μm^2^ x 1000 in arbitrary units (AU). The red bars indicate the average ±SEM. ****P<0.0001; one way ANOVA with Dunnett post test. **(B)** Immunoblot analysis of CPL-1* degradation in *edem-1* and *edem-2* mutants carrying the rescuing EDEM-1::mCherry and EDEM-2::mCherry transgenes, respectively. Total protein lysates derived from *edem-1* and *edem-2* mutants carrying the CPL-1* and rescuing transgenes were separated by SDS-PAGE and immunoblotted with anti-GFP polyclonal antisera. Tubulin was used as loading control. **(C)** Histogram showing the densitometry values of the bands presented in (B) normalized to the value of WT condition (n = 3 ± SEM, one way ANOVA with Fisher test), *P<0.05; **P<0.01).
(DOCX)

S3 Fig EDEM depletion mitigates ER stress. **(A)** Representative confocal images of WT(EV) control or indicated RNAi depleted young adults carrying the P*hsp-4*::GFP transgene under non ER-stress conditions (upper panel), treatment with 5 μg/ml tunicamycin for 12h (middle panel), and heat shock, and 5 μM thapsigargin treatment (lower panel). TM-tunicamycin, HS-heat shock, TG-thapsigargin. The worms were outlined for better visualization. Images were obtained using the same confocal settings. Exposure adjustments were uniformly applied. Scale bar: 50 μm. **(B)** Quantitative RT-PCR measurements of P*hsp-4*::mRNA levels in indicated strains under non stress and treatment with 5 μg/ml tunicamycin (n = 3 independent experiments). **(C)** Quantitative RT-PCR measurements of P*hsp-4*::mRNA levels in indicated strains under non stress and heat stress treatment (n = 3 independent experiments). In **(B)** and **(C)** quantifications were normalized relative to WT non stress conditions. ****P<0.0001, *ns*, not significant. Quantifications were normalized relative to WT non stress conditions. **(D)** Representative confocal images of WT control and *edem-2* mutants carrying the PC12C8.1::GFP transgene under non ER-stress conditions. The left panels show fluorescence images and the right panels DIC images.
(DOCX)

S4 Fig *edem* response to thapsigargin. **(A)** Quantitative RT-PCR measurements of *edem* mRNA levels in WT young animals under non stress (NS) and treatment with 5 μM thapsigargin (TG), (n = 3 independent experiments). **(B)** Percentage of eggs that developed into L4 larvae after 3 days on 10 μM thapsigargin. Each strain was scored in three independent experiments in triplicates. **(C)** Quantification of WT (EV) or RNAi-treated day one adults carrying P*hsp-4*::GFP transgene, treated or not with 5 μM thapsigargin. The worms were kept on RNAi plates for one generation before exposure to ER stress treatments. Values represent mean fluorescence/μm^2^ x 1000. The red bars indicate the average ±SEM. **(D)** Analysis of *xbp-1* spliced/unspliced ratio from young adults of indicated strains. Total RNA was isolated, reverse transcribed and used for PCR analysis of *xbp-1* splicing forms.
(DOCX)

S1 Table Strains used in this study. (DOCX)

S2 Table Primers used in this study. (DOCX)

S3 Table Raw numerical data of all the figures. (XLSX)
