# Supplementary material for: Targeting EDEM protects against ER stress and improves development and survival in C. elegans
Source: PLoS Genet. 2022 Feb 22;18(2):e1010069. doi: 10.1371/journal.pgen.1010069 (PMC8912907; doi:10.1371/journal.pgen.1010069)
Supplement: S1 Fig — (A) Quantitative real-time PCR (qPCR) of edem-3 mRNA. WT and edem-3 mutant worms treated with empty vector or edem-3 RNAi; expression were normalized to that of cdc-42 and pmp-3. (B) Total protein lysates derived from WT Pedem::GFP transgenic animals subjected to the indicated treatment were separated by SDS-PAGE and immunoblotted with anti-GFP polyclonal antisera; tubulin was used as loading control. NS- non-treated, TM- tunicamycin, HS-heat stress, OS- osmotic stress. The histograms show the densitometry values of Pedem::GFP bands normalized to the value of of NS condition (n = 3 ± SEM, t test), *P<0.05; **P<0.01; ***P<0.001; ns, not significant. (C) RNAi downregulation of edem triggered accumulation of CPL-1* in intestinal cells. To overrule a significant contribution of autofluorescent stress granules to the GFP fluorescence, images captured with Diode laser were included. Scale bar: 20 μm. (DOCX) [file pgen.1010069.s001.docx]

**
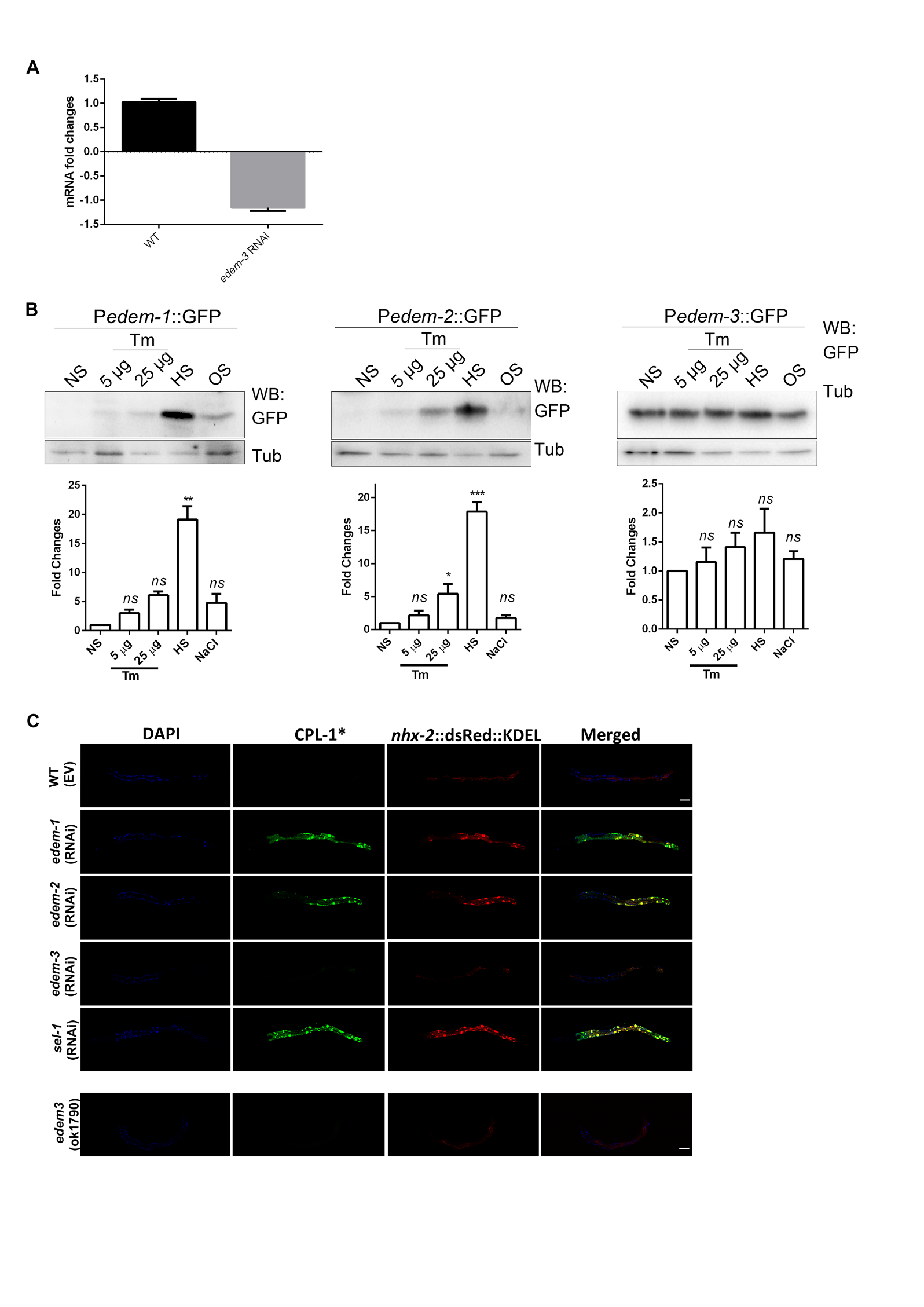
S1 Fig. Immunoblot analysis of P*edem*::GFP expression in young animals.**  **(A)** Quantitative real-time PCR (qPCR) of *edem-3* mRNA. WT and *edem-3* mutant worms treated with empty vector or *edem-3* RNAi; expression were normalized to that of *cdc-42* and *pmp-3*. **(B)** Total protein lysates derived from WT P*edem*::GFP transgenic animals subjected to the indicated treatment were separated by SDS-PAGE and immunoblotted with anti-GFP polyclonal antisera; tubulin was used as loading control. NS- non-treated, TM- tunicamycin, HS-heat stress, OS- osmotic stress. The histograms show the densitometry values of P*edem*::GFP bands normalized to the value of of NS condition (n=3 ± SEM, *t* test),  **P*<0.05; ***P*<0.01; ****P*<0.001; *ns*, not significant. **(C)** RNAi downregulation of *edem* triggered accumulation of CPL-1* in intestinal cells. To overrule a significant contribution of autofluorescent stress granules to the GFP fluorescence, images captured with Diode laser were included. Scale bar: 20 μm.
