# Supplementary material for: Targeting EDEM protects against ER stress and improves development and survival in C. elegans
Source: PLoS Genet. 2022 Feb 22;18(2):e1010069. doi: 10.1371/journal.pgen.1010069 (PMC8912907; doi:10.1371/journal.pgen.1010069)
Supplement: S2 Fig — (A) Fluorescence intensities of WT animals carrying the CPL-1* transgene treated with indicated concentrations of kifunensine. Values in scatter gram represent mean fluorescence/μm2 x 1000 in arbitrary units (AU). The red bars indicate the average ±SEM. ****P<0.0001; one way ANOVA with Dunnett post test. (B) Immunoblot analysis of CPL-1* degradation in edem-1 and edem-2 mutants carrying the rescuing EDEM-1::mCherry and EDEM-2::mCherry transgenes, respectively. Total protein lysates derived from edem-1 and edem-2 mutants carrying the CPL-1* and rescuing transgenes were separated by SDS-PAGE and immunoblotted with anti-GFP polyclonal antisera. Tubulin was used as loading control. (C) Histogram showing the densitometry values of the bands presented in (B) normalized to the value of WT condition (n = 3 ± SEM, one way ANOVA with Fisher test), *P<0.05; **P<0.01). (DOCX) [file pgen.1010069.s002.docx]

**
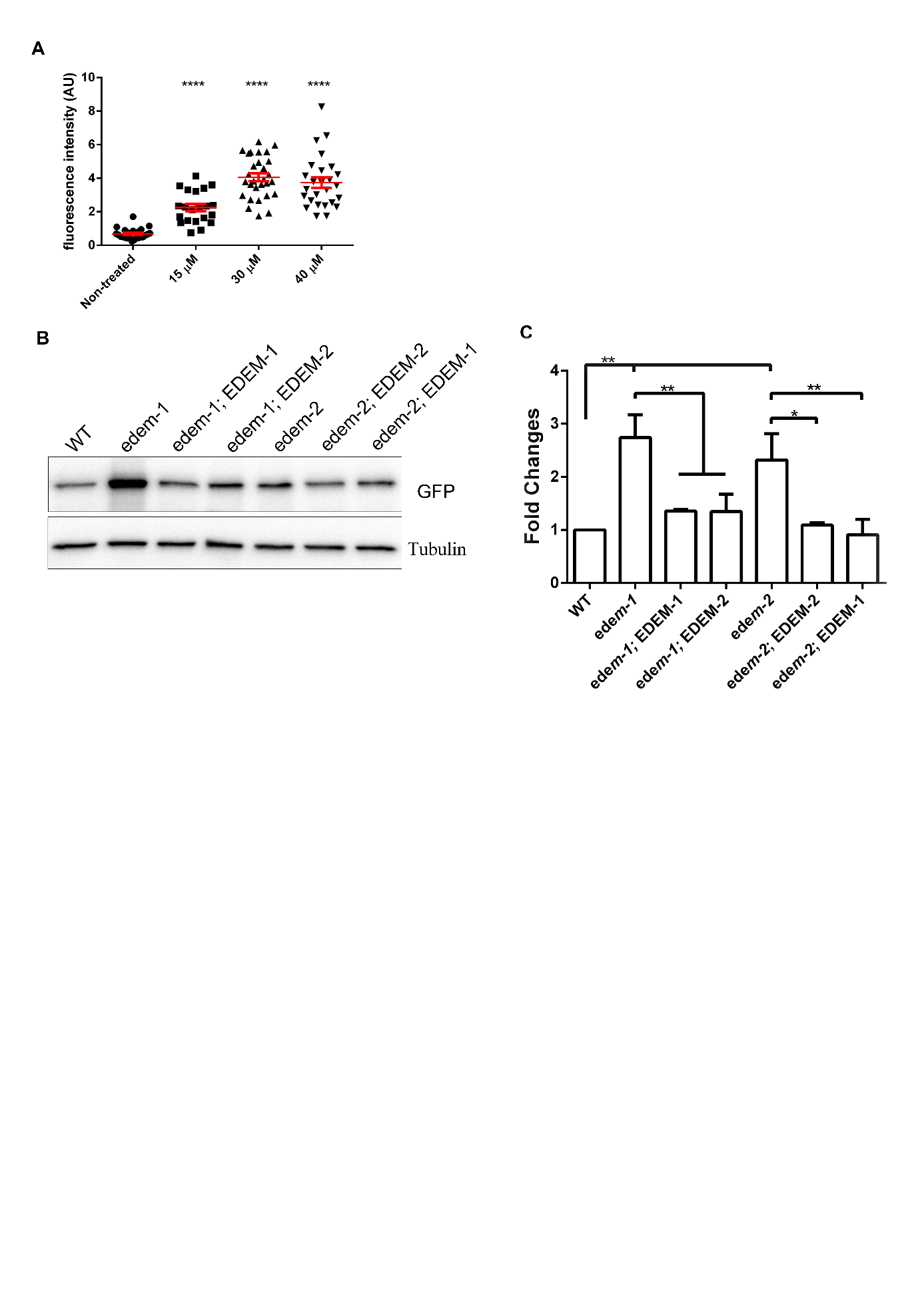
**

**S2 Fig. EDEM-1 and EDEM-2 are required for CPL-1* degradation** **(A)** Fluorescence intensities of WT animals carrying the CPL-1* transgene treated with indicated concentrations of kifunensine. Values in scatter gram represent mean fluorescence/µm^2^ x 1000 in arbitrary units (AU). The red bars indicate the average ±SEM. *****P*<0.0001; one way ANOVA with Dunnett post test. **(B)** Immunoblot analysis of CPL-1* degradation in *edem-1* and *edem-2* mutants carrying the rescuing EDEM-1::mCherry and EDEM-2::mCherry transgenes, respectively. Total protein lysates derived from *edem-1* and *edem-2* mutants carrying the CPL-1* and rescuing transgenes were separated by SDS-PAGE and immunoblotted with anti-GFP polyclonal antisera. Tubulin was used as loading control. **(C)** Histogram showing the densitometry values of the bands presented in (B) normalized to the value of WT condition (n=3 ± SEM, one way ANOVA with Fisher test),  **P*<0.05; ***P*<0.01).
