# Supplementary material for: Targeting EDEM protects against ER stress and improves development and survival in C. elegans
Source: PLoS Genet. 2022 Feb 22;18(2):e1010069. doi: 10.1371/journal.pgen.1010069 (PMC8912907; doi:10.1371/journal.pgen.1010069)
Supplement: S3 Fig — (A) Representative confocal images of WT(EV) control or indicated RNAi depleted young adults carrying the Phsp-4::GFP transgene under non ER-stress conditions (upper panel), treatment with 5 μg/ml tunicamycin for 12h (middle panel), and heat shock, and 5 μM thapsigargin treatment (lower panel). TM-tunicamycin, HS-heat shock, TG-thapsigargin. The worms were outlined for better visualization. Images were obtained using the same confocal settings. Exposure adjustments were uniformly applied. Scale bar: 50 μm. (B) Quantitative RT-PCR measurements of Phsp-4::mRNA levels in indicated strains under non stress and treatment with 5 μg/ml tunicamycin (n = 3 independent experiments). (C) Quantitative RT-PCR measurements of Phsp-4::mRNA levels in indicated strains under non stress and heat stress treatment (n = 3 independent experiments). In (B) and (C) quantifications were normalized relative to WT non stress conditions. ****P<0.0001, ns, not significant. Quantifications were normalized relative to WT non stress conditions. (D) Representative confocal images of WT control and edem-2 mutants carrying the PC12C8.1::GFP transgene under non ER-stress conditions. The left panels show fluorescence images and the right panels DIC images. (DOCX) [file pgen.1010069.s003.docx]

**
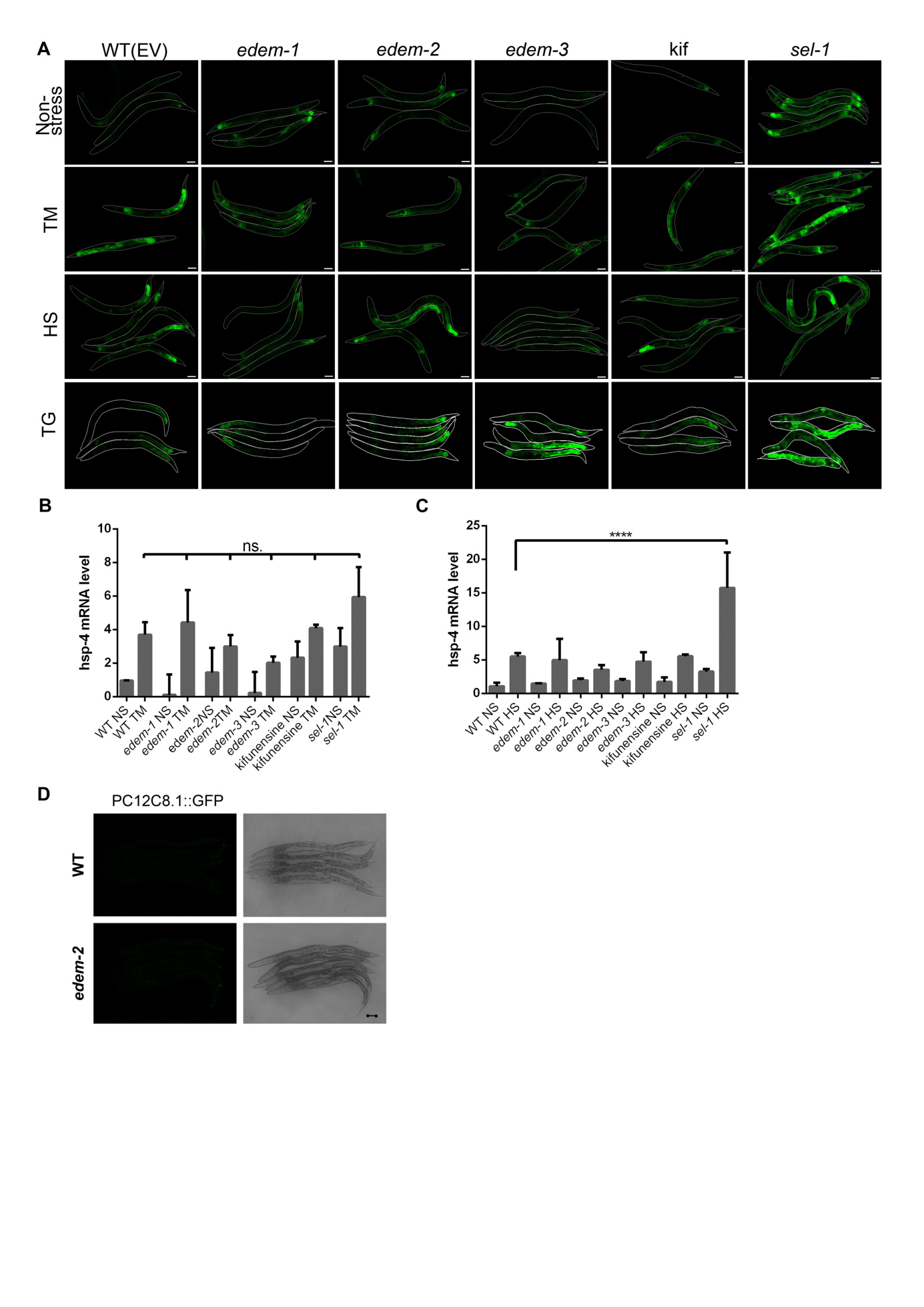
**

**S3 Fig. EDEM depletion mitigates ER stress. (A)** Representative confocal images of WT(EV) control or indicated RNAi depleted young adults carrying the P*hsp-4*::GFP transgene under non ER-stress conditions (upper panel), treatment with 5 μg/ml tunicamycin for 12h (middle panel), and heat shock, and 5 μM thapsigargin treatment (lower panel). TM-tunicamycin, HS-heat shock, TG-thapsigargin. The worms were outlined for better visualization. Images were obtained using the same confocal settings. Exposure adjustments were uniformly applied. Scale bar: 50 μm. **(B)** Quantitative RT-PCR measurements of  P*hsp-4*::mRNA levels in indicated strains under non stress and treatment with 5 μg/ml tunicamycin (n = 3 independent experiments). **(C)** Quantitative RT-PCR measurements of  P*hsp-4*::mRNA levels in indicated strains under non stress and heat stress treatment (n = 3 independent experiments). In **(B)** and **(C)** quantifications were normalized relative to WT non stress conditions. *****P*<0.0001, *ns*, not significant. Quantifications were normalized relative to WT non stress conditions.  **(D)** Representative confocal images of WT control and *edem-2* mutants carrying the PC12C8.1::GFP transgene under non ER-stress conditions. The left panels show fluorescence images and the right panels DIC images.
