# Supplementary material for: Targeting EDEM protects against ER stress and improves development and survival in C. elegans
Source: PLoS Genet. 2022 Feb 22;18(2):e1010069. doi: 10.1371/journal.pgen.1010069 (PMC8912907; doi:10.1371/journal.pgen.1010069)
Supplement: S4 Fig — (A) Quantitative RT-PCR measurements of edem mRNA levels in WT young animals under non stress (NS) and treatment with 5 μM thapsigargin (TG), (n = 3 independent experiments). (B) Percentage of eggs that developed into L4 larvae after 3 days on 10 μM thapsigargin. Each strain was scored in three independent experiments in triplicates. (C) Quantification of WT (EV) or RNAi-treated day one adults carrying Phsp-4::GFP transgene, treated or not with 5 μM thapsigargin. The worms were kept on RNAi plates for one generation before exposure to ER stress treatments. Values represent mean fluorescence/μm2 x 1000. The red bars indicate the average ±SEM. (D) Analysis of xbp-1 spliced/unspliced ratio from young adults of indicated strains. Total RNA was isolated, reverse transcribed and used for PCR analysis of xbp-1 splicing forms. (DOCX) [file pgen.1010069.s004.docx]

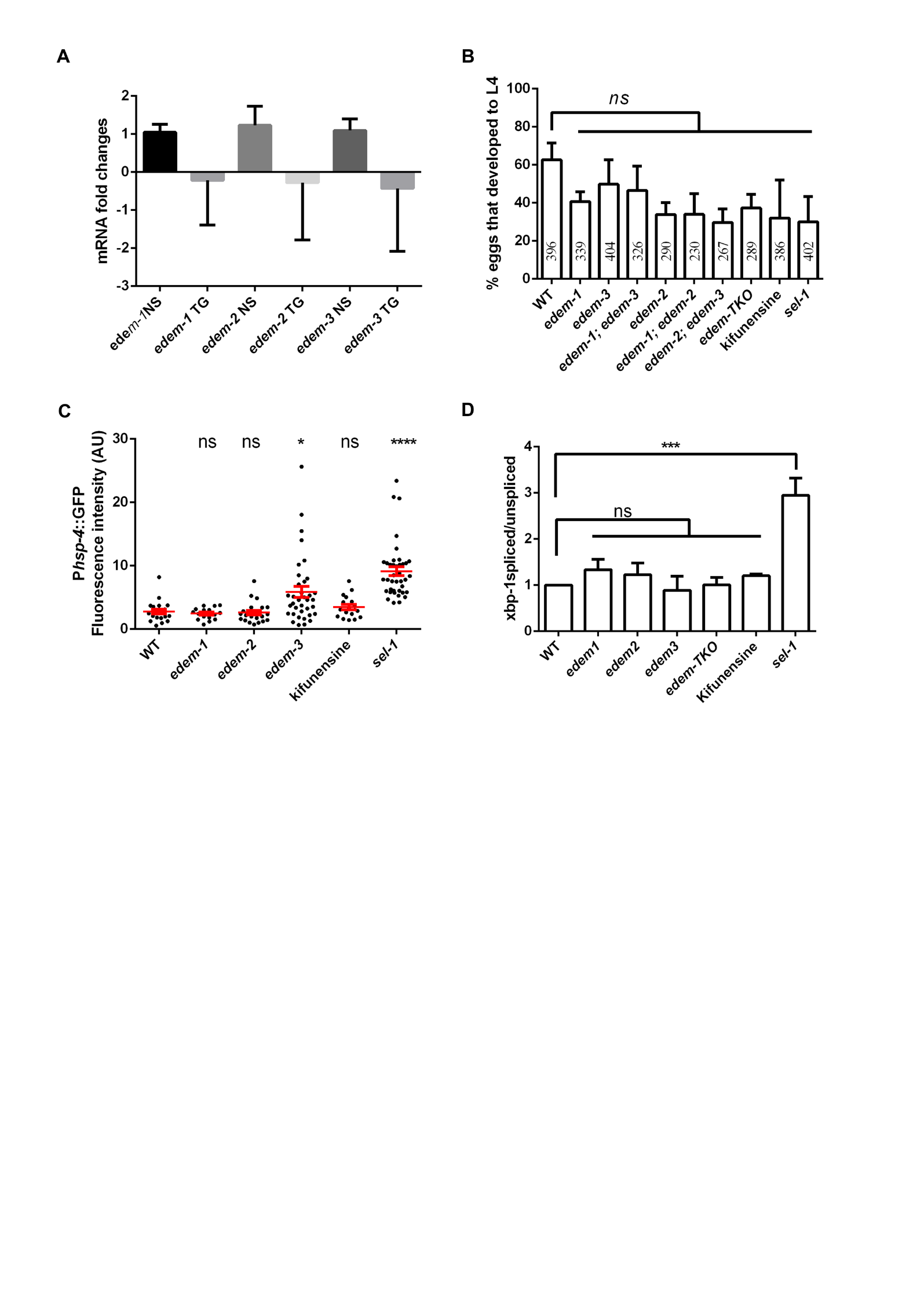
**S4 Fig.** *edem* response to thapsigargin. **(A)** Quantitative RT-PCR measurements of  *edem* mRNA levels in WT young animals under non stress (NS) and treatment with 5 μM thapsigargin (TG), (n = 3 independent experiments).  **(B)** Percentage of eggs that developed into L4 larvae after 3 days on 10 μM thapsigargin. Each strain was scored in three independent experiments in triplicates. **(C)** Quantification of WT (EV) or RNAi-treated day one adults carrying P*hsp-4*::GFP transgene, treated or not with 5 μM thapsigargin. The worms were kept on RNAi plates for one generation before exposure to ER stress treatments. Values represent mean fluorescence/µm^2^ x 1000. The red bars indicate the average ±SEM. **(D)** Analysis of *xbp-1* spliced/unspliced ratio from young adults of indicated strains. Total RNA was isolated, reverse transcribed and used for PCR analysis of *xbp-1* splicing forms.
