# Supplementary material for: Targeting EDEM protects against ER stress and improves development and survival in C. elegans
Source: PLoS Genet. 2022 Feb 22;18(2):e1010069. doi: 10.1371/journal.pgen.1010069 (PMC8912907; doi:10.1371/journal.pgen.1010069)
Supplement: S1 Table — (DOCX) [file pgen.1010069.s005.docx]

**S1 Table. Strains used in this study.**

| **Strain** | **Genotype** | **Source** | **Notes:** |
| --- | --- | --- | --- |
| N2 | *wild type* | CGC |  |
| IBC105 | *sgEx1 [Pedem-1::GFP, rol-6(su1006)]* | This study |  |
| IBC104 | *sgEx2 [Pedem-2::GFP, rol-6(su1006)]* | This study |  |
| IBC94 | *sgEx3 [Pedem-3::GFP, rol-6(su1006)]* | This study |  |
| IBC107 | *sgEx4 [Pedem-1::EDEM-1::GFP, rol-6(su1006)]* | This study |  |
| IBC95 | *sgEx4 [Pedem-1::EDEM-1::mCherry, rol-6(su1006)]* | This study |  |
| IBC106 | *sgEx5 [Pedem-2::EDEM-2::GFP, rol-6(su1006)]* | This study |  |
| IBC96 | *sgEx5 [Pedem-2::EDEM-2::mCherry, rol-6(su1006)]* | This study |  |
| VK 1258 | *vkEx1258 [nhx-2p::cpl-1(W32AY35A)::YFP + nhx-2p::DsRed::KDEL].* | CGC | [1] |
| *RE666* | *ire-1 (v33)II* | CGC | [2] |
| IBC456 | *edem-1(tm5068)IV* | NBRP | Outcrossed 8x |
| IBC395 | *edem-2(tm5186)V* | NBRP | Outcrossed 12x |
| *VC1301* | *edem-3(ok1790)X* | CGC | Outcrossed 8x |
| IBC574 | *sel-1(tm3901)V* | NBRP | Outcrossed 8x |
| PD4793 | *mIs10 [myo-2p::GFP + pes-10p::GFP + gut-promoter::GFP] V.* | CGC | Outcrossed >20x |
| PD4792 | *mIs11 [myo-2p::GFP + pes-10p::GFP + gut-promoter::GFP] IV* | CGC | Outcrossed >20x |
| IBC460 | *edem-1(tm5068)IV; edem-3(ok1790)X* | This study |  |
| IBC643 | *edem-1(tm5068)IV; edem-2(tm5186)V* | This study |  |
| IBC477 | *edem-2(tm5186)V; edem-3(ok1790)X* | This study |  |
| IBC541 | *edem-1(tm5068)IV; edem-2(tm5186)V; edem-3(ok1790)X* | This study |  |
| VK1879 | *vkEx1879 [nhx-2p::cpl-1(W32A Y35A)::YFP + myo-2p::mCherry].* | CGC | [3] |
| IBC543 | *edem-1(tm5068)IV; vkEx1879 [nhx-2p::cpl-1(W32A Y35A)::YFP + myo-2p::mCherry]* | This study |  |
| IBC432 | *edem-2(tm5186)V; vkEx1879 [nhx-2p::cpl-1(W32A Y35A)::YFP + myo-2p::mCherry]* | This study |  |
| IBC452 | *edem-3(ok1790)X; vkEx1879 [nhx-2p::cpl-1(W32A Y35A)::YFP + myo-2p::mCherry]* | This study |  |
| IBC566 | *edem-1(tm5068)IV; edem-3(ok1790)X; vkEx1879 [nhx-2p::cpl-1(W32A Y35A)::YFP + myo-2p::mCherry]* | This study |  |
| IBC577 | *edem-1(tm5068)IV; edem-2(tm5186)V; vkEx1879 [nhx-2p::cpl-1(W32A Y35A)::YFP + myo-2p::mCherry]* | This study |  |
| IBC563 | *edem-2(tm5186)V; edem-3(ok1790)X; vkEx1879 [nhx-2p::cpl-1(W32A Y35A)::YFP + myo-2p::mCherry]* | This study |  |
| IBC678 | *edem-1(tm5068)IV; edem-2(tm5186)V; edem-3(ok1790)X; vkEx1879 [nhx-2p::cpl-1(W32A Y35A)::YFP + myo-2p::mCherry]* | This study |  |
| IBC565 | *sel-1(tm3901); vkEx1879 [nhx-2p::cpl-1(W32A Y35A)::YFP + myo-2p::mCherry]* |  |  |
| IBC583 | *edem-1(tm5068IV); sgEx4 [Pedem-1::EDEM-1::mCherry, rol-6(su1006)]; vkEx1879 [nhx-2p::cpl-1(W32A Y35A)::YFP + myo-2p::mCherry]* | This study |  |
| IBC580 | *edem-1(tm5068IV); sgEx5 [Pedem-2::EDEM-2::mCherry, rol-6(su1006)]; vkEx1879 [nhx-2p::cpl-1(W32A Y35A)::YFP + myo-2p::mCherry]* | This study |  |
| IBC560 | *edem-2(tm5186)V; sgEx5 [Pedem-2::EDEM-2::mCherry, rol-6(su1006)]; vkEx1879 [nhx-2p::cpl-1(W32A Y35A)::YFP + myo-2p::mCherry]* | This study |  |
| IBC582 | *edem-2(tm5186)V; sgEx4 [Pedem-1::EDEM-1::mCherry, rol-6(su1006)]; vkEx1879 [nhx-2p::cpl-1(W32A Y35A)::YFP + myo-2p::mCherry]* | This study |  |
| VS25 | *hjIs14 [vha-6p::GFP::C34B2.10(SP12) + unc-119(+)]X* | CGC | [4] |
| IBC579 | *sgEx4 [Pedem-1::EDEM-1::mCherry, rol-6(su1006)]; hjIs14 [vha-6p::GFP::C34B2.10(SP12) + unc-119(+)]X* | This study |  |
| IBC578 | *sgEx5 [Pedem-2::EDEM-2::mCherry, rol-6(su1006)]; hjIs14 [vha-6p::GFP::C34B2.10(SP12) + unc-119(+)]X* | This study |  |
| SJ4005 | *zcIs4 [hsp-4::GFP]V* | CGC | [5] |
| VS30 | *hjSi158 [vha-6p::SEL-1(1-79)::mCherry::HDEL::let-858 3'UTR ]I.* | CGC | [6] |
| IBC464 | *edem-1(tm5068)IV; hjSi158[vha-6p::SEL-1(1-79)::mCherry::HDEL::let-858 3'UTR ]I.* | This study |  |
| IBC423 | *edem-2(tm5186)V; hjSi158 [vha-6p::SEL-1(1-79)::mCherry::HDEL::let-858 3'UTR ]I.* | This study |  |
| IBC425 | *edem-3(ok1790)X; hjSi158 [vha-6p::SEL-1(1-79)::mCherry::HDEL::let-858 3'UTR ]I.* | This study |  |
| IBC550 | *sel-1(tm3901)V; hjSi158 [vha-6p::SEL-1(1-79)::mCherry::HDEL::let-858 3'UTR ]I.* | This study |  |
| RB545 | *pek-1(ok275)X* | CGC | outcrossed 6x |
| RB772 | *atf-6(ok551)X* | CGC | outcrossed 6x |
| ICB 566 | *edem-3(ok1790)X; ire-1(v33)* | This study |  |
| ICB 567 | *edem-2(tm5186)V; ire-1(v33)* | This study |  |
| ICB 569 | *edem-2(tm5186)V; xbp-1(zc12)* | This study |  |
| ICB 592 | *edem-2(tm5186)V; pek-1(ok275)X* | This study |  |
| ICB 593 | *edem-2(tm5186)V; atf-6(ok551)X* | This study |  |

References

1. Mark T. Miedel, Nathan J. Graf, Kate E. Stephen, Olivia S. Long, Stephen C. Pak, David H. Perlmutter, Gary A. Silverman, Cliff J. Luke. [A Pro-Cathepsin L Mutant Is a Luminal Substrate for Endoplasmic-Reticulum-Associated Degradation in C. elegans](https://www.ncbi.nlm.nih.gov/pmc/articles/PMC3388072/). PLoS One. 2012; 7(7): e40145; doi: 10.1371/journal.pone.0040145
2. [X Shen](https://pubmed.ncbi.nlm.nih.gov/?sort=pubdate&term=Shen+X&cauthor_id=11779465)^,^ [R E Ellis](https://pubmed.ncbi.nlm.nih.gov/?sort=pubdate&term=Ellis+RE&cauthor_id=11779465), [K Lee](https://pubmed.ncbi.nlm.nih.gov/?sort=pubdate&term=Lee+K&cauthor_id=11779465), [C Y Liu](https://pubmed.ncbi.nlm.nih.gov/?sort=pubdate&term=Liu+CY&cauthor_id=11779465), [K Yang](https://pubmed.ncbi.nlm.nih.gov/?sort=pubdate&term=Yang+K&cauthor_id=11779465), [A Solomon](https://pubmed.ncbi.nlm.nih.gov/?sort=pubdate&term=Solomon+A&cauthor_id=11779465), [H Yoshida](https://pubmed.ncbi.nlm.nih.gov/?sort=pubdate&term=Yoshida+H&cauthor_id=11779465), [R Morimoto](https://pubmed.ncbi.nlm.nih.gov/?sort=pubdate&term=Morimoto+R&cauthor_id=11779465), [D M Kurnit](https://pubmed.ncbi.nlm.nih.gov/?sort=pubdate&term=Kurnit+DM&cauthor_id=11779465), [K Mori](https://pubmed.ncbi.nlm.nih.gov/?sort=pubdate&term=Mori+K&cauthor_id=11779465), [R J Kaufman](https://pubmed.ncbi.nlm.nih.gov/?sort=pubdate&term=Kaufman+RJ&cauthor_id=11779465). Complementary signaling pathways regulate the unfolded protein response and are required for C. elegans development. Cell, 2001;107(7):893-903;doi: 10.1016/s0092-8674(01)00612-2.
3. Miedel MT, Graf NJ, Stephen KE, Long OS, Pak SC, Perlmutter DH, Silverman GA, Luke CJ. A Pro-Cathepsin L Mutant Is a Luminal Substrate for Endoplasmic-Reticulum-Associated Degradation in *C. elegans.* PLoS ONE 7(7): e40145. <https://doi.org/10.1371/journal.pone.0040145>.
4. Xu N, Zhang SO, Cole RA, McKinney SA, Guo F, Haas JT, Bobba S, Farese RV Jr, Mak HY.The FATP1-DGAT2 complex facilitates lipid droplet expansion at the ER-lipid droplet interface, J Cell Biol 2012;198(5):895-911. doi: 10.1083/jcb.201201139.
5. Calfon M, Zeng H, Urano F, Till JH, Hubbard SR, Harding HP, Clark SG, Ron D. IRE1 couples endoplasmic reticulum load to secretory capacity by processing the XBP-1 mRNA. Nature. 2002 ; 415(6867):92-6. doi: 10.1038/415092a.
6. Klemm RW, Norton JP, Cole RA, Li CS, Park SH, Crane MM, Li L, Jin D, Boye-Doe A, Liu TY, Shibata Y, Lu H, Rapoport TA, Farese RV Jr, Blackstone C, Guo Y, Mak HY. A conserved role for atlastin GTPases in regulating lipid droplet size. Cell Rep. 2013;3(5):1465-75. doi: 10.1016/j.celrep.2013.04.015.
