# Supplementary material for: Targeting EDEM protects against ER stress and improves development and survival in C. elegans
Source: PLoS Genet. 2022 Feb 22;18(2):e1010069. doi: 10.1371/journal.pgen.1010069 (PMC8912907; doi:10.1371/journal.pgen.1010069)
Supplement: S2 Table — (DOCX) [file pgen.1010069.s006.docx]

**S2 Table. Primers used in this study.**

| **Gene** | **Forward primer** | **Reverse primer** | **Notes** |
| --- | --- | --- | --- |
| *edem-1* | CGGAGGAGAGCTGACGTGTCACTATTC | GATTCCAGGGGCTTGAATCTGCACAATG | Used for genotyping |
| *edem-2* | CCTTGCTTCCAGGCACCTACTAAATGAA | TTGAAAGCAGGCATAAGTCGATCAGC | Used for genotyping |
| *edem-3* | GAGTGTTGCAGTGTGTGTGCGTGC GGAGATGTTGACGATGTCCTGGGAAAGTG | CGAGAAGAGAGTCCATGAAGCCCCTTG | Used for genotyping |
| *sel-1* | ATGATTAAAACCTATCTGACACTGTTGCTAC | GTTTCGTCGGTTAGTCTCAATCGTTG | Used for genotyping |
| *xbp-1* | GCATCTACCAGAACGTCGTCTACG | TCCATACGACGGAGTTGGTTGCTG | Used for *xbp-1* splicing |
| *edem-1* | GATCGATTCTCTTCAGGCAAGTTTCG | GCATAGTAAAGGGCATGATGGCACAC | Used for RT-PCR |
| *edem-2* | GGAATGTTGGATTGCTGTAGTGTACAAGC | CTCATCACCATTGTTCACTTGTTTCACACC | Used for RT-PCR |
| *edem-3* | CATCAAGAAATTGTTGAGCGTCACGTC | TGGAACGCATAGTCAGCATCCTTG | Used for RT-PCR |
| *pmp-3* | GAGAACTTGCTGGAGTCACTCATCGTG | CTGAAGATGGTACTGTAGATGGAGGACG | Used for RT-PCR |
| *hsp-4* | CTACTTCAACGGCAAGGAGC | CACCTCCGATTACTCCTGCT | Used for RT-PCR |
| *cdc-42* | TATCGTATCCACAGACCGACGTGTTTC | CAGGCACCCATTTTTCTCGGACATTC | Used for RT-PCR |
